# Tetramethylalloxazines as efficient singlet oxygen photosensitizers and potential redox-sensitive agents

**DOI:** 10.1038/s41598-023-40536-4

**Published:** 2023-08-17

**Authors:** Anna Golczak, Dorota Prukała, Ewa Sikorska, Mateusz Gierszewski, Volodymyr Cherkas, Dorota Kwiatek, Adam Kubiak, Naisargi Varma, Tomasz Pędziński, Shaun Murphree, Radek Cibulka, Lucyna Mrówczyńska, Jacek Lukasz Kolanowski, Marek Sikorski

**Affiliations:** 1 Faculty of Chemistry, Adam Mickiewicz University, Uniwersytetu Poznańskiego 8, 61-614 Poznan, Poland; 2 Poznań University of Economics and Business, Al. Niepodległości 10, 61-875 Poznan, Poland; 3 Faculty of Physics, Adam Mickiewicz University, Uniwersytetu Poznańskiego 2, 61-614 Poznan, Poland; 4 Institute of Bioorganic Chemistry, Polish Academy of Sciences, Noskowskiego 12/14, 61-704 Poznan, Poland; 5 Department of Chemistry, Allegheny College, 520 N. Main Street, Meadville, PA USA; 6 Department of Organic Chemistry, University of Chemistry and Technology, Prague, Technicka 5, 16628 Prague 6, Czech Republic; 7 Faculty of Biology, Adam Mickiewicz University, Uniwersytetu Poznańskiego 6, 61-614 Poznan, Poland

**Keywords:** Photochemistry, Physical chemistry

## Abstract

Tetramethylalloxazines (TMeAll) have been found to have a high quantum yield of singlet oxygen generation when used as photosensitizers. Their electronic structure and transition energies (S_0_ → S_i_, S_0_ → T_i_, T_1_ → T_i_) were calculated using DFT and TD-DFT methods and compared to experimental absorption spectra. Generally, TMeAll display an energy diagram similar to other derivatives belonging to the alloxazine class of compounds, namely *π*,π* transitions are accompanied by closely located n,π* transitions. Photophysical data such as quantum yields of fluorescence, fluorescence lifetimes, and nonradiative rate constants were also studied in methanol (MeOH), acetonitrile (ACN), and 1,2-dichloroethane (DCE). The transient absorption spectra were also analyzed. To assess cytotoxicity of new compounds, a hemolytic assay was performed using human red blood cells (RBC) in vitro. Subsequently, fluorescence lifetime imaging experiments (FLIM) were performed on RBC under physiological and oxidative stress conditions alone or in the presence of TMeAll allowing for pinpointing changes caused by those compounds on the intracellular environment of these cells.

## Introduction

**Figure 1:**
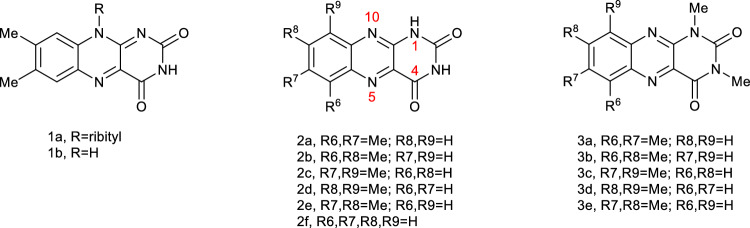
The isoalloxazine class (**1**) versus the alloxazine class (**2**, **3**) of compounds. Alloxazines **3a**-**3d** are the subject of the present study.

Isoalloxazines (10-substituted tetrahydro-3*H*,10*H*-benzo[g]pteridine-2,4-diones), specifically flavins (7,8-dimethylsubstituted isoalloxazines), continue to be a subject of ongoing research due to their widespread biological activity and presence in nature. Flavins (**1**, Fig. 1), serve as coenzymes or receptors in a variety of biochemical reactions. As a result of their nutritional significance and abundance in nature, flavins are among the most extensively studied classes of compounds. In contrast, alloxazines (tetrahydro-1*H*,3*H*-benzo[*g*]pteridine-2,4-diones), which are closely related to isoalloxazines, have received comparatively little attention. Initially, interest in the properties of alloxazines, particularly lumichrome (**2e**), was primarily driven by their association with isoalloxazines and their identification as photodecomposition products of flavins. However, recent studies have highlighted the significance of alloxazines in biological systems and their potential as useful molecular probes.

**Figure 2:**
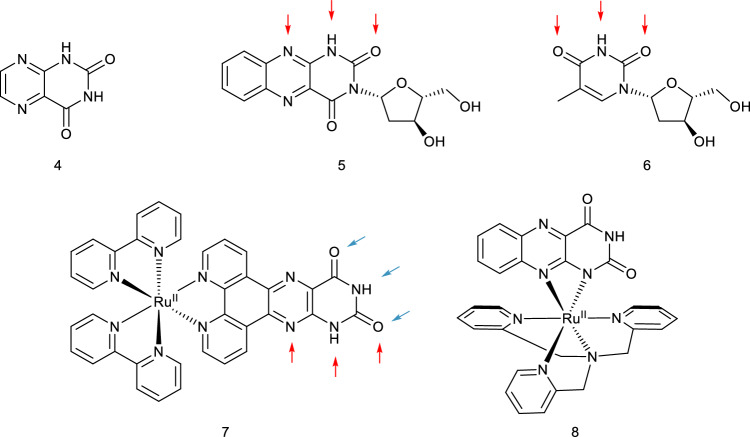
Top: Lumazine (**4**), alloxazine-3-nucleoside (**5**), thymidine (**6**), with arrows showing relevant H-bonding sites for DNA base recognition. Bottom: DNA intercalating ruthenium-pteridinophenanthrolinedione complex (**7**) with arrows showing the duplicate H-bonding patterns, and a photoisomerizable ruthenium-alloxazine complex (**8**).

Synthetic alloxazine analogues of lumichrome (**2e**) have demonstrated potential as biological probes due to their ability to selectively bind adenine in DNA duplexes, exhibiting similar hydrogen bond patterns to those found in DNA. It makes them useful as fluorescent probes. In particular, the heterocyclic lumazine core (**4**, Fig. 2) of alloxazine (**2f**) selectively binds adenine in DNA duplexes^1^, and alloxazine-3-nucleosides (for example **5**) exhibit the same hydrogen bonding pattern as thymidine (**6**)^2^. In a similar vein, the ruthenium(II)-bis(bipyridine) complex of pteridinophenanthrolinedione (**7**) can act as a “light switch” upon intercalation with duplex DNA, and its exceptionally strong binding is attributed to a hydrogen bond pattern similar to the interactions involved in triplex DNA formation^3^. Another kind of “light-switch” behavior is observed with the ruthenium(II)-tris(2-pyridylmethyl)amine complex with alloxazine (**8**), which undergoes rapid isomerization by pseudo-rotation of the alloxazine ligand after irradiation with light between 400–600 nm^4^.

Lumichrome (**2e**) is an endogenous chromophore in many organisms, including humans^5^. However, high levels are deleterious to cells, so sophisticated mechanisms have evolved to prevent the accumulation of phototoxic levels of **2e**. An example is the use of a protein called dodecin, which binds to **2e** and eliminates it through degradation in certain prokaryotes^6^. In particular, dodecin is believed to promote the degradation of riboflavin (**1a**) to lumichrome (**2e**) in *Archea*^7^; thus, **2e** is a key intermediate in the complex mechanism of flavin homeostasis. Furthermore, **2e** has been found to exhibit discrete signaling behavior in organisms and has been identified as an inducer of larval metamorphosis in the ascidian *Halocynthia roretzi*^8,9^. Also, the photoactive nature of **2e** makes it an interesting candidate for photodynamic therapies. For example, treatment with a dilute **2e** solution followed by irradiation in the 340–440 nm range resulted in a reduction of more than 99% in *E. faecalis* cultures^5^; moreover, **2e** has a generally good safety profile^10^.

**2e** and its alloxazine analogues have potential applications beyond just biological systems. For example, **2e** forms very strong complexes with fluoride and acetate, allowing reliable spectrophotometric titration of these analytes in acetonitrile (ACN), even in the presence of other anions such as chloride, bromide, iodide, chlorate, and thiocyanate^11^. Alloxazines are also efficient singlet oxygen photosensitizers^12–14^, which can be used for wastewater remediation applications. Studies have shown that **2e** is more effective than titanium dioxide in the photodegradation of various phenol derivatives, and polychlorophenols can be completely dechlorinated in 30 min using artificial sunlight^15^.

Alloxazines (e.g. **2e**) can undergo rapid tautomerization when irradiated under certain conditions, particularly in protic environments to the corresponding isoalloxazine **1b**^16–21^ through an excited state double proton transfer (ESDPT) process. This photoinduced proton lability has inspired the use of **2e** as a novel optical transistor material^22^. When thin films of **2e** are deposited on conductive SnO_2_ glass and illuminated at 365 nm, the current through the material is enhanced through a photoinduced proton transfer effect, which is amplified by the presence of acetic acid. This property has been explored for potential applications in optoelectronics^22^.

As part of our ongoing research on alloxazine photophysics and photochemistry,^23–25^, calculations and experimental studies have been performed on alloxazines with monomethyl^23^, dimethyl^26^, and trimethyl^27^ carbocyclic moieties (i.e., methyl substituents at C6-C9). These findings, and intensive work by others, especially recent papers by Mroginski and Miller^28,29^, indicate that the substituent pattern has important effects on the solubility and spectroscopic parameters of these derivatives.

Substitution at N1 shuts down the ESDPT process, and consequently N1-alkyl derivatives can engage in photochemistry without the confounding effect of tautomerization to flavins, even in protic medium. The photochemical properties of some substituted alloxazines have been studied in the context of photocatalysis^30–32^. Thus, N1,N3-dialkylalloxazines represent an interesting entry into versatile light-stable photocatalysts. We began to characterize this class of alloxazines by examining those with a third methyl group on the carbocyclic ring^27^, and in the present study we have extended the scope to tetramethyl derivatives (Fig. 1, right), with a particular interest in comparing their spectroscopic and photophysical properties to the corresponding N1,N3-unsubstituted analogs^26^.

To investigate the potential biological applications of new compounds as redox-sensitive agents, their hemocompatibility was first evaluated by measuring hemolytic activity against human red blood cells (RBC) in vitro. In experimental studies, human RBC are widely used as a convenient cell model for assessing the cytotoxicity of chemical compounds^33,34^. As has been demonstrated in our previous studies, RBC are used both in evaluating the antioxidant activity of newly synthetized compounds^35^ and in assessing their potential utility as redox-sensitive agents^36,37^. To induce oxidative stress inside the RBC, the hydrophobic free radical generator *tert*-butyl hydroperoxide (TB) was added to the cell suspension. To analyze the redox status of RBC, FLIM analysis was performed. This research may have important implications for the development of new compounds with potential practical applications.

## Experimental

### Materials

A series of tetramethylalloxazines (**3a-3d**, Fig. 1) were available from previous work, and synthetized as described elsewhere^38^. MeOH and other solvents of spectroscopic or HPLC grades (Aldrich, Merck) were used as received.

### Steady-state spectral and photophysical measurements

Steady-state fluorescence spectra were obtained using a Jobin Yvon-Spex Fluorolog 3–221 spectrofluorometer and UV–visible absorption spectra were obtained using a Shimadzu UV-2550 spectrophotometer. Fluorescence quantum yield of compounds **3a**-**3d** have been determined by preparing series of dilution ranging in absorption from 0.02 to 0.10 and excited at their respective absorption maxima. **2e** has been used as standard in experiment to determine fluorescence quantum yield^39^. For **2e** the excitation wavelength 378 nm have been used. The following equation have been applied to determine the quantum yield of fluorescence:

$${\Phi }_{{\text{x}}} = {\Phi }_{{{\text{ST}}}} \left( {{\raise0.7ex\hbox{${{\text{Grad}}_{{\text{x}}} }$} \!\mathord{\left/ {\vphantom {{{\text{Grad}}_{{\text{x}}} } {{\text{Grand}}_{{{\text{ST}}}} }}}\right.\kern-0pt} \!\lower0.7ex\hbox{${{\text{Grand}}_{{{\text{ST}}}} }$}}} \right)\left( {\frac{{{\upeta }_{{\text{X}}}^{2} }}{{{\upeta }_{{{\text{ST}}}}^{2} }}} \right)$$

where ST stand for the standard, X stand for the respective **3a**-**3d** derivative, Φ_ST_ is the quantum yield of standard, Φ_X_ is the quantum yield of respective **3a**-**3d** derivative, Grad is the gradient from the plot of integrated fluorescence intensity versus optical density, η is refractive index of the solvent.

Time-resolved fluorescence measurements were performed using a system that has previously been described in detail^40^. The system includes a frequency-doubled output of a mode-locked, synchronously pumped, cavity-dumped argon-ion/DCM dye-laser for excitation, and a Hamamatsu microchannel plate photomultiplier coupled to a single-photon timing system for detection. Emission excitation matrix was collected with the A-TEEMs™ (absorbance-transmission and fluorescence excitation-emission matrix) method using a Horiba Scientific Aqualog spectrophotometer. Aqualog integrates its data acquisition functions directly with the Origin software. All EEMs data were corrected for the influence of inner filter effects (IFE) and Rayleigh masking.

### Laser flash photolysis

The laser flash photolysis (LFP) setup used in this work has been described in detail elsewhere^41^. Samples for LFP experiments were excited using 355 nm, the third harmonic of an Nd: YAG laser (Quantel, model Q-smart 450) with pulses of 6–8 ns duration and energy kept at approximately 3 mJ/pulse (to avoid unnecessary multi-photonic processes). The monitoring system consisted of a 150 W pulsed Xe lamp (Hamamatsu, model E7536, Japan), a monochromator (Princeton Instruments, model Spectra Pro SP-2155, USA), and an R955 model photomultiplier (Hamamatsu, Japan), powered by a PS-310 power supply (Stanford Research System, Sunnyvale, CA, USA). The data processing system consisted of real-time acquisition using a digital oscilloscope (Tektronix, model MDO 3024, 350 MHz, USA) which was triggered by a fast photodiode (Thorlabs, DET10M, ~ 1 ns rise time). The data from the oscilloscope were transferred to a computer equipped with software based on LabView 8.0 (National Instruments, Austin, TX, USA) which controls the timing and acquisition functions of the system. Kinetic traces were taken between 350 and 700 at 5–10 nm intervals. The respective compounds **3a**-**3d** solutions were deoxygenated by bubbling argon for 15 min prior to measurements. The experiments were also conducted in air-equilibrated solution. The experiments were carried out in square (1 cm × 1 cm) fused-silica cells.

### Determination of quantum yields of singlet oxygen production (Ф_Δ_)

The singlet oxygen quenching was monitored directly via the time-resolved O_2_^(1^Δ_g_) → O_2_(^3^Σ_g_^−^) phosphorescence, using a FluoTime 300 spectrometer (PicoQuant, Germany) equipped with a Peltier-cooled H10330-45 NIR detector (Hamamatsu, Japan). Time-resolved measurements were conducted utilizing the "burst mode" functionality of the spectrometer, also known as the Multi-Channel Scaling (MCS) method^42^. In this mode of FluoTime 300 operation, multiple laser pulses (typically 5.

500 pulses at a 40 MHz repetition rate from a LDH-405 diode laser operated at 408 nm, or a LDH-375 diode laser operated at 378 nm) were used to excite a dissolved sensitizer. ^1^O_2_ phosphorescence decays were adequately modeled using a single exponential fitting function yielding values of the ^1^O_2_ lifetime.

The singlet oxygen quantum yields were determined using the gradient method by preparing a series of dilutions. Measurements were recorded on the Pico Quant-Fluo Time 300 fluorescence lifetime spectrometer with an H10330B-45 NIR-PMT module, which is sensitive in the 950 to 1400 nm NIR range with excitation (λ_exc_ = 408 nm, or 378 nm) using perinaphthenone as standard (Φ_Δ_ = 0.95, λ_exc_ = 408 nm). The excitation source for steady-state emission spectra was a xenon lamp with a monochromator. Phosphorescence ^1^O_2_ decays were measured using a pulsed laser diode head (λ_exc_ = 408 nm, or 378 nm) at around λ_em_ = 1270 nm on the same spectrofluorometer.

Quantum yields of singlet oxygen production (Ф_Δ_) generated in the presence of TMeAll in organic solvents (acetonitrile, (ACN), 1,2-dichloroethane (DCE) and methanol, (MeOH)) were calculated using the results of steady-state measurements. Air-equilibrated solutions of the TMeAll were optically matched (+ /- 0.001 absorbance units) at the excitation wavelength (380 nm) to a standard reference solution. Perinaphthenone (Per) in the same solvents was used as a reference, as it has Ф_Δ_ = Φ_ST_ = 0.95 ± 0.05, independent of the solvent used^43,44^.

Two sets of solutions with absorption optical density maxima between 0.02 and 0.10 at the excitation wavelength were prepared, one containing TMeAll and the other containing Per in the same solvent. The solutions were excited at their respective absorption maxima, and the characteristic emission spectra of singlet oxygen were recorded for each substance. The total area under the emission spectrum was calculated separately for each substance. Finally, the quantum yield of singlet oxygen (Φ_Δ_) was calculated by comparing the total area under the emission spectrum of TMeAll and Per according to the equation:

$$\Phi_{{\text{x}}} = \Phi_{{{\text{ST}}}} \left[ {\frac{{{\text{Grad}}_{{\text{X}}} }}{{{\text{Grad}}_{{{\text{ST}}}} }}} \right]$$

Here, Φ_X_ is the quantum yield of the singlet oxygen production by tetramethylalloxazine; Φ_ST_ is the same for the standard sample (Per); Grad_X_ and Grad_ST_ are the slopes of the graphs of the integrated singlet oxygen intensity versus optical density of TMeAll and Per, respectively.

### Theoretical procedures

#### TD-DFT Calculations

Information on the electronic structure and geometry of TMeAll was obtained using quantum-chemical calculations by means of density functional theory (DFT). To allow direct comparison, the calculations were performed exactly the same way as in our earlier publication devoted to monomethyl substituted alloxazines derivatives^23^. That is, the calculations were performed using the B3LYP functional^45,46^ in conjunction with a modest split-valence polarized basis set of 6-31G (d)^47^. The B3LYP functional is one of the empirical hybrid density functionals devised by Becke^45^, which combines a fraction of the Hartree–Fock exchange functional with the correlation functional of Lee, Yang and Parr^46^. The functional contains 3 parameters determined by fitting to selected properties of a large set of molecules. The B3LYP functional is widely used in the calculation of electronic transitions^48^. The oscillator strengths were calculated in the dipole length representation. The Gaussian 03 package of ab initio programs was used for calculations of excitation energies and oscillator strengths^49^.

We used the optimized ground state geometry for calculations predicting lowest-energy singlet–singlet S_0_ → S_i_ transitions as well as spin-forbidden S_0_ → T_i_ transitions presented for TMeAll, whereas T_1_ → T_i_ excitation energies and transition intensities were determined for the optimized geometry of the lowest triplet state (T_1_), using the unrestricted method suitable for open-shell systems. To verify the type of minimum located, geometry optimization was followed by vibrational frequency calculations, which revealed no imaginary frequencies. No attempts were made to locate alternative minima using different starting structures. We compare the theoretical data obtained with the B3LYP/6-31G(d) methods with the experimental absorption spectra. It can be concluded that the accuracy is 2000–3000 cm^−1^, usually requiring a shift towards the blue to reproduce experimental spectra, see Fig. 1S.

### Biological part

#### Hemocompatibility studies of 3a-3d

##### Reagents

All reagents were purchased from Avantor Performance Materials Poland SA (Gliwice, Poland). Standard oxidant *tert-*butyl hydroperoxide (**TB**) was purchased from Sigma-Aldrich Chemie.

##### Human red blood cells (RBC)

As mentioned and described earlier in references^36,37^, fresh human red blood cells (RBC) suspensions as concentrates (hematocrit = 65%) were purchased from the blood bank in Poznań, according to the bilateral agreement no. ZP/2867/D/21 between the Blood Bank and Adam Mickiewicz University in Poznań. The RBC suspension was washed three times (3000 rpm, 10 min, + 4 °C) in 7.4 pH buffered phosphate saline (PBS – 137 mM NaCl, 2.7 mM KCl, 10 mM Na_2_HPO_4_, 1.76 mM KH_2_PO_4_) supplemented with 10 mM glucose. After washing, RBC were suspended in PBS buffer supplemented with 10 mM glucose at 1.65 × 10^9^ cells/mL (hematocrit = 15%), stored at + 4 °C and used within 5 h.

##### In vitro hemolytic activity assay

The hemolytic potency of **3a-3d** was determined by a standard hemolytic assay^50^. Briefly, RBC (1.65 × 10^8^ cells/mL, hematocrit = 1.5%) were incubated in PBS buffer (7.4 = pH) supplemented with 10 mM glucose and containing compounds tested at a concentration equal to 0.1 mg/mL for 1 h and for 24 h at 37 °C in a thermoshaker (BioSan Thermo-Shaker TS-100 C, Biosan, Riga, Latvia). For the positive and negative controls, the RBC were incubated in deionized ice-cold water or in PBS buffer, respectively. Each sample was tested in triplicate and the experiments were repeated with RBC obtained from three different blood donors. After incubation, the RBC suspensions were washed with PBS buffer (3000 rpm, 10 min at 4 °C) (Sigma 3–30 K Sartorious AG, Gottingen, Germany) and the degree of hemolysis was estimated by measuring the absorbance values (Ab) of the sample supernatant at *λ* = 540 nm using a BioMate™ 160 UV–Visible spectrophotometer (Thermo Scientific, Waltham, MA, USA). The results were expressed as the percentage (%) of hemolysis, which was determined using the following equation:

Hemolysis (%) = (Ab sample tested/Ab positive control) × 100.

where the positive control is the absorbance (Ab) of supernatant obtained after incubation of RBC in ice-cold water. The results are presented as the mean values (n = 9) ± standard deviation (SD).

##### RBC preparation for fluorescence‑lifetime imaging microscopy (FLIM) analysis

RBC (1.65 × 10^8^ cells/mL, hematocrit = 1.5%) were incubated in PBS buffer (7.4 = pH) supplemented with 10 mM glucose (control cells) and with the compounds tested at a final concentration of 0.1 mg/mL for 24 h at 37 °C (final DMSO concentration < 0.1%) in a thermoshaker (BioSan Thermo-Shaker TS-100 C, Biosan, Riga, Latvia). After incubation, RBC were washed three times (3000 rpm, 10 min, 4 °C) (Sigma 3–30 K Sartorious AG, Gottingen, Germany). To induce oxidative stress conditions in the RBC, *tert-*butyl hydroperoxide (TB), a standard free radical inducer, was added to a final concentration of 250 μM. RBC were then incubated with and without TB for 1.5 h at 37 °C with gentle shaking. Each sample was tested in triplicate, and the experiments were repeated three times with RBC obtained from different blood donors. After incubation, all RBC suspensions were washed three times with PBS buffer (3000 rpm, 10 min, 4 °C), (Sigma 3–30 K Sartorious AG, Gottingen, Germany). The prepared RBC suspensions were subsequently used for FLIM analysis.

##### Fluorescence‑lifetime imaging microscopy (FLIM) analysis of RBC

Following the previously described procedure^36^, the RBC suspensions (80 µL) were placed on microscope slides and covered with microscopy cover slips. A large number of intact RBC were studied in several separate experimental samples using an Abberior combined MINFLUX/STED superresolution laser scanning confocal microscopy system (Abberior Instruments, Germany). The system is equipped with pulsed lasers, high-sensitivity photon counting avalanche photodiode (APD) detectors in tunable spectral configuration, and a dual channel time correlated single photon counting (TCSPC) system (PicoQuant, Germany). The microscope is built on an Olympus IX83 chassis (Olympus, Japan). To minimize image aberrations caused by refractive index mismatch, a water immersion objective Olympus 60 × NA 1.2 UPLSAPO60XW (Olympus, Japan) was used with an optimized coverslip thickness correction collar. Two independent spectral detection channels were used to acquire fluorescence lifetime information simultaneously. The instrument response function (IRF) full width half maximum (FWHM) was about 200 ps for the complete instrument. The modulation frequency was set to 80 MHz. The TCSPC parameters were as follows: a 25 ns detection range and 1000 bins. Spectral settings for channels were selected based on fluorescent emission spectra acquired at the same microscope, with all settings identical except for the emission channel. After optimization, identical emission windows were used for all experimental conditions, with fluorescence emission split into two channels: 420 to 550 nm and 550 to 780 nm. FLIM was applied to study compounds **3a-3d** in RBC under normal (PBS buffer) and oxidative stress conditions (250 µM TB in PBS for 1.5 h, following the above protocol). All imaging experiments were performed at room temperature.

The following abbreviations for the differently treated RBC samples will be used from now on:

**RBC** – control cells (in PBS buffer),

**RBC** + **TB** – **RBC** incubated with 250 μM TB in PBS buffer (1.5 h, 37 °C),

**RBC** + **X** – **RBC** incubated with 0.1 mg/mL X (24 h, 37 °C) in PBS buffer (where **X** is the alloxazine compound, **3a**-**3d**),

**RBC** + **X** + **TB** – **RBC** preincubated with 0.1 mg/mL **X** (24 h, 37 °C) in PBS buffer and then incubated with 250 µM TB (1.5 h, 37 °C).

The brightness and color-scales on representative cell images correspond to the number of photon counts per pixel and the averaged fluorescence lifetime per pixel (without IRF subtraction). The results and fluorescence lifetime distribution histograms from at least three images per each condition (each image composed of 100 consecutively recorded frames, with each image typically containing more than 100 single cells) were analyzed using SymPhoTime64 software (PicoQuant, Germany). The instrument response function (IRF) was subtracted prior to data fitting.

### Statistical analysis

For the hemolytic activity, data were plotted as mean values ± standard deviation (SD) of the results of three independent experiments, with every sample at least in triplicate (n = 9). Statistical analyses included factorial one-way ANOVA followed by Tukey’s honest significant difference (HSD) test at α = 0.05 performed using the SPSS statistical analysis.

## Results and discussion

### Theoretically predicted and experimental gas-phase spectra

A significant number of iso- and alloxazines, including monomethyl substituted iso- and alloxazines, and dimethyl-, trimethyl- and tetramethyl isoalloxazines, have been studied using similar DFT and TD-DFT calculations for singlet and triplet absorption spectra^23,24,39,51–54^. The results from the TD-DFT^24,39,51,55,56^ for iso- and alloxazines have shown some improvements compared to previous semi-empirical and ab initio calculations^57,58^. In particular, previously published results have successfully reproduced the expected configuration of singlet excited states and the oscillator strengths of the respective transitions. In the present study, TD-DFT calculations were performed using 6-31G(d) to ensure consistency with previously published results.

This approach has been further validated by a study carried out by Neiss et al.^51^ in which an extensive computational study of flavin-related molecules was performed. The TD-DFT (B3LYP) methods were applied using basis sets ranging from small, without polarization or diffuse functions (6-31G, 80 basis functions), to large (aug-cc-pVTZ, 460 basis functions) to calculate the ten lowest singlet and the ten lowest triplet excited states above the geometry-optimized singlet ground state of B3LYP/6-31G* geometry. They found that reasonably accurate results can be obtained even with medium-sized basis sets such as 6-31G*. This conclusion is consistent with our own results for not only flavins but also for alloxazines. In particular, we have made calculations with larger basis sets for several iso- and alloxazines, comparing 6-31G(d) and 6-311G(d, p), and also using the PCM method to account for the MeOH solvent (see, for example, the results obtained in^59^), arriving at similar conclusions.

Although no gas-phase spectroscopic data have been reported for the compounds treated in the present investigation, it is instructive to examine the trends for two representative tetramethyl derivatives for which data are available, namely 1,3-dimethyllumichrome (**3e**) and 3,7,8,10-tetramethylisoalloxazine (**9**), and to compare the absorption of these compounds in the gas phase and in a solvent, 1,4-dioxane. We found (see Fig. 1S) that the position of the lowest energy absorption maximum is relatively unchanged by the solvent and is in good agreement with the gas phase and solvent values. For example, the corresponding absorption for 1,3-dimethyllumichrome (**3e**) is 26.5 × 10^3^ cm^−1^ in the gas phase^60^ and 26.18 × 10^3^ cm^−1^ in 1,4-dioxane^27^. Similarly good agreement is found with 3,7,8,10-tetramethylisoalloxazine (**9**), with energies of 22.9 × 10^3^ cm^−1^ and 22.5 × 10^3^ cm^−1^ in the gas phase^60^ and in 1,4-dioxane^27^. The lowest energy transitions for both compounds are of *π*, π* character at approximately 27.5 × 10^3^ cm^−1^ for **3e** and 24.5 × 10^3^ cm^−1^ for **9**^27^. The computed oscillator strengths confirm that only the *π*, π* transitions should be observable. These results strongly suggest that the position of the absorption bands should not be significantly affected by the change from the gas phase to the solution phase, although some impact on the position and shape of the absorption bands by strongly interacting solvents cannot be ruled out.

We acknowledge an erroneous claim in our earlier work on isoalloxazines and alloxazines^55^ that gas phase spectra were unavailable for the compounds studied, and we thank Prof. Frank Müller for pointing out this oversight and making us aware of the corresponding data^60–62^.

### Tetramethylalloxazines (TMeAll): singlet states

As shown in Table 1 and Fig. 1S (Supporting Information), the four TMeAll in the study exhibit a characteristic property of alloxazines and other aza-aromatic compounds, where the two lowest-energy *π*,π* transitions are accompanied by closely located n,π* transitions. This leads to the *proximity effect*^63^. Furthermore, **3a** is predicted to have the largest energy separation of the two singlet states, with |*E*_n,π*_ − *E*_*π*,π*_|= 800 cm^−1^. Similar close-neighboring n,π* and *π*,π* states appear for the predicted S_0_ → S_3_ and S_0_ → S_4_ transitions, with the largest calculated energy separation of the two singlet states |*E*_n,π*_—*E*_*π*,π*_|= 900 cm^−1^ for **3b** and |*E*_n,π*_—*E*_*π*,π*_|= 800 cm^−1^ for **3d**. The position of the methyl group (see data in Table 1 and Fig. 1S) was found to have a significant impact on the energy of the π,π* transitions, while the n,π* transitions are relatively unaffected.

**Table 1:** The lowest predicted (B3LYP /6-31G(d)) S_0_ → S_i_ excitation energies of tetramethylalloxazines with their corresponding oscillator strengths, *f*.

S_0_ → S_i_	**3a**		**3b**		**3c**		**3d**	
	*E* × 10^–3^/cm^−1^	*f*	*E* × 10^–3^/cm^−1^	*f*	*E* × 10^–3^/cm^−1^	*f*	*E* × 10^–3^/cm^−1^	*f*
→ S_1_	26.4	0.045	27.4^b^	0.001	26.9	0.066	27.2	0.037
→ S_2_	27.2^b^	0.001	27.6	0.034	27.4^b^	0.001	27.3^b^	0.001
→ S_3_	31.2	0.168	30.5	0.220	31.4^b^	< 0.001	30.6	0.208
→ S_4_	31.3^b^	< 0.001	31.4^b^	< 0.001	31.5	0.134	31.4^b^	< 0.001
→ S_5_	35.8^b^	0.003	35.9^b^	0.003	35.9^b^	0.004	35.9^b^	0.004
→ S_6_	38.6^b^	0	39.2^b^	0	38.6^b^	0	38.6^b^	0
→ S_7_	39.0	0.206	38.7	0.142	39.2	0.346	39.5^b^	0
→ S_8_	39.5^b^	0	39.3^b^	0	39.3^b^	0	39.5	0.241
→ S_9_	40.5^b^	0	40.8^b^	0	40.8^b^	< 0.001	40.5^b^	< 0.001
→ S_10_	41.4	0.560	41.3	0.326	41.1	0.414	41.4	0.372
→ S_11_	42.8	0.391	42.7	0.671	42.8	0.364	42.8	0.498
→ S_12_	44.5^b^	0	44.1^b^	< 0.001	44.0^b^	< 0.001	44.3^b^	< 0.001
→ S_13_	46.9	0.057	47.4^b^	0	47.0	0.042	47.1	0.080
→ S_14_	47.7^b^	< 0.001	47.7^b^	< 0.001	47.4	0.025	47.6^b^	< 0.001
→ S_15_	47.8	0.015	47.8	0.073	47.8^b^	< 0.001	47.7^b^	0.007

b-n,π* state, otherwise *π*,π* state.

Figure 2S illustrates the shape of the lowest unoccupied molecular orbital (LUMO), the highest occupied molecular orbital (HOMO), and HOMO-2 involved in the transitions to low-lying excited states of **3b** and **3c**. The S_0_ → S_1_ transition in **3b** has a dominant contribution from the HOMO-2 → LUMO excitation and is assigned as an n,π* transition, which is accompanied by closely located *π*,π* transitions with a dominant contribution from the HOMO → LUMO excitation. On the other hand, in the cases **3a**, **3c**, and **3d,** the lowest energy transition predicted for these derivatives has a dominant contribution from the HOMO → LUMO excitation and is assigned as an *π*,π* transition. In the case of **3a**, **3c**, and **3d**, the S_0_ → S_2_ transition has a dominant contribution from the HOMO-2 → LUMO excitation and corresponds to an n,π* transition.

**Figure 3:**
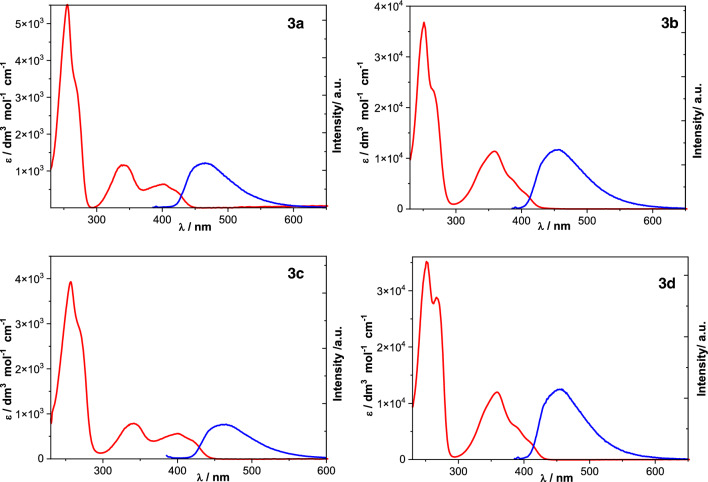
Absorption spectra (red line) and emission spectra (blue line) of **3a**, **3b**, **3c** and **3d** in 1,2-dichloroethane.

These calculations agree well with the experimental results. As shown in Fig. 3, the absorption spectra taken in DCE (red line) exhibit two absorption bands in the lower energy region for all four derivatives, with the positions of the absorption maxima varying according to the substitution pattern. The observation of two maxima in the experiment matches the theoretical predictions. Note that the longest wavelength absorption band in **3b** and **3d** is not as distinct.

**Figure 4:**
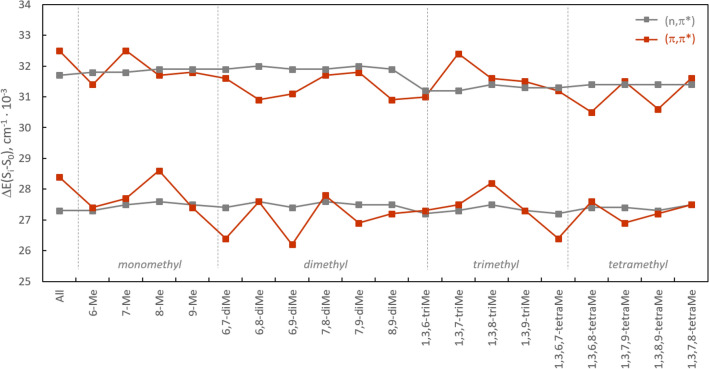
Predicted lowest singlet–singlet transitions of alloxazine derivatives. The two calculated lowest-energy transitions in alloxazines presented are of the *π*,π* character, located at approximately 31 700 cm^−1^ and 27 400 cm^−1^, and are accompanied, respectively, by two closely located n,π* transitions of low oscillator strengths.

It is informative to compare the TMeAll (**3**) with their N1,N3-unsubstituted counterparts (i.e., **2**), for which the experimental and theoretical data are summarized in Tables 1S and 2S. As shown in Fig. 4, the addition of methyl substituents at N1 and N3 has little impact on the predicted lowest-level singlet–singlet (*π*, π*) transitions, and only a modest decrease of the next-lowest-level (*π*, π*) transitions, which accounts for the slight red shift in the observed absorption for TMeAll (**3**) vs. their N1,N3 unsubstituted counterparts (**2**).

As the above calculation and experimental results show, the spectral properties of TMeAll are virtually the same as those of the corresponding N1,N3 unsubstituted alloxazines **2** (Tabl3 1S). The only noticeable difference is the presence of an additional n,π* transition at about 35.9 × 10^3^ cm^−1^ in the predicted S_0_ → S_i_ transitions for, which is absent in dimethylalloxazines (**2**).

Finally, to provide a full context, Fig. 4 summarizes the predicted lowest singlet–singlet transitions for various monomethyl, dimethyl, trimethyl, and tetramethyl- substituted alloxazines (a total of 20 derivatives). Some trends are evident, namely, generally the n,π* and *π*,π* singlet excited states are close to each other in all the compounds examined, with Δ*E* < 1200 cm^−1^, and in some cases these are practically isoenergetic. In six of the alloxazine derivatives, the lowest-lying transition is *π*, π*; in eleven of the compounds, the *π*,π* and n,π* transitions are practically isoenergetic; and three examples have a lowest-lying transition that is n,π*.

The experimental absorption spectra taken in MeOH, ACN, and DCE (see Fig. 3) exhibit two absorption bands in the lower energy region for all four derivatives, with the positions of the absorption maxima varying according to the substitution pattern. The results are presented in Table 2. Considerable variation is observed in the molar absorption coefficients (ε) among the derivatives and the solvent used. For example, the molar absorption coefficient for λ_2_, (ε_2_) in MeOH is substantially higher for **3b** and **3d** (11,620 and 11,870 dm^3^ mol^−1^ cm^−1^, respectively) than for **3a** and **3c** (600 and 1690 dm^3^ mol^−1^ cm^−1^, respectively).

**Table 2:** Spectroscopic and photophysical data for the singlet states of tetramethylalloxazines.

Solvent	Comp	λ_2_ /nm (ε_2_/M^−1^ cm^−1^)	λ_1_ /nm (ε_1_/M^−1^ cm^−1^)	λ_F_ /nm	Φ_F_	τ_F_ /ns	*k*_r_ /10^8^ s^−1^	Σ*k*_nr_ /10^8^ s^−1^	Φ_Δ_	τ_T_ /µs
1,2-DCE	**3a**	341 (1161)	400 (643)	465	0.047	1.37	0.34	6.96	0.93	
	**3b**	359 (11,378)	–	458	0.018	0.78	0.23	12.6	0.65	
	**3c**	342 (788)	400 (564)	466	0.046	1.26	0.36	7.57	0.84	
	**3d**	359 (11,984)		457	0.017	0.98	0.17	10.0	0.77	
ACN	**3a**	338 (1040)	397 (612)	466	0.057	1.88	0.30	5.02	0.85	22.3
	**3b**	355 (10,351)	–	456	0.021	0.92	0.23	10.6	0.90	11.7
	**3c**	335 (751)	397 (614)	465	0.047	1.49	0.32	6.40	0.86	15.3
	**3d**	354 (10,387)	–	457	0.020	0.77	0.26	12.7	0.86	20.3
MeOH	**3a**	338 (1686)	393 (931)	481	0.062	3.54	0.18	2.65	0.97	
	**3b**	357 (11,618)	–	459	0.024	0.62	0.39	15.7	0.96	23.2
	**3c**	338 (599)	396 (456)	474	0.077	2.98	0.26	3.10	0.78	16.3
	**3d**	358 (11,866)	–	471	0.042	1.98	0.21	4.84	0.98	26.1

λ_1_, λ_2_ are the positions of the two lowest-energy bands in the absorption spectra, λ_F_ the fluorescence emission maximum, Φ_F_ the fluorescence quantum yield, τ_F_ the fluorescence lifetime, *k*_r_ the radiative rate constant and Σ*k*_nr_ the sum of nonradiative rate constants, Ф_Δ_ the quantum yield of photosensitized production of singlet oxygen, τ_T_ the triplet lifetime.

The absorption bands (λ_1_ and λ_2_) of TMeAll in DCE, ACN, and MeOH (Table 2) are red-shifted vs. the corresponding dimethylalloxazines **2**^23,26^. The shifts are largest in DCE (Δλ_2_ = 6–9 nm and Δλ_1_ = 20–25 nm) and are rather small in MeOH (Δλ_2_ = 3–5 nm and Δλ_1_ = 2–7 nm).

Fluorescence emission spectra for dimethyl- with respect to the tetramethyl- substituted alloxazines show a single band in all solvents examined, with the position of the emission maximum varying depending on solvent and substitution pattern. This is illustrated in Fig. 3, which shows the spectra in DCE (spectra in other solvents are not shown). In the case of dimethylalloxazines^26^, the emission spectra in 1,4-dioxane, DCE, and ACN are very similar, but in MeOH solutions, a red shift of emission maxima is observed. For the 7-substituted derivatives (**3a** and **3c**), the fluorescence maxima are not significantly shifted (Δλ_F_ = 1–3 nm) vs. their counterparts (**2a** and **2c**), while the 8-substituted series (**3b** and **3d** vs. **2b** and **2d**) exhibit a much more pronounced shift (Δλ_F_ = 5–16 nm), with the largest shift occurring in MeOH. Thus, fluorescence maxima show little dependence on solvent polarity per se, but hydrogen bonding results in a shift of the emission maximum wavelength.

Other parameters are also affected, such as fluorescence quantum yield, lifetime, and radiative and nonradiative rates. For example, fluorescence quantum yields are higher for TMeAll vs. dimethylalloxazines in DCE and ACN; but in MeOH solutions, the opposite is generally true (e.g., 0.075 for **2a** vs. 0.062 for **3a**), corresponding to extended lifetimes of dimethylalloxazine excited states in all of the solvents used. Fluorescence lifetimes were shorter for TMeAll vs. dimethylalloxazines in all solvents used, and in general fluorescence lifetimes for both dimethyl and tetramethyl derivatives were relatively short in solvents such as ACN and DCE, but longer in MeOH (Table 2).

Additionally, methyl substitution at specific positions can extend or shorten the fluorescence quantum yields and lifetimes. For example, the 8-substituted derivatives (i.e., **3b,d**) exhibit relatively lower fluorescence quantum yields and shorter lifetimes in all solvents compared to the 7-substituted derivatives (i.e., **3a,c**). These differences are the result of variations in rates of nonradiative decay, and the C8 methyl group plays a pivotal role in modulating these processes. Similar results are observed for the dimethyl and monomethyl derivatives^23,26^. It is well established that excited-state double proton transfer (ESDPT) occurs in the presence of acetic acid for alloxazine derivatives giving isoalloxazinic derivatives, and other molecules have also been implicated in this process, including water^64–67^, alcohols^19,65,68^, and pyridine^17,69^. However, these intriguing reports still need further studies to generate consensus. In any event, the substitution of the methyl group at N1 shuts down this pathway.

All excited alloxazines exhibit relatively short fluorescence decay times in ACN and DCE; however, longer fluorescence lifetimes are observed in MeOH. The values of *k*_r_ and Σ*k*_nr_ (Table 2) show that, as is typical for most alloxazines, the singlet state decay is dominated by nonradiative processes, and the rates of radiative and nonradiative decay are reduced in protic solvents. The reductions in both radiative and nonradiative rates are also observed in MeOH, with nonradiative rates perturbed more strongly, leading to longer fluorescence lifetimes and slightly higher fluorescence quantum yields.

The introduction of methyl groups at the carbocyclic positions (C6-C9) has previously been shown to affect the spectral and photophysical characteristics of alloxazines^23^, yet substitution at N1 and/or N3 has almost no effect, as seen in the example of **2e** vs its N1,N3 dimethyl analogue^39^. Similarly, no noticeable effect on spectral and photophysical properties was observed by methyl substitution at N3 for isoalloxazines, as seen in lumiflavin vs. its 3-methyl derivative^39^.

### Singlet oxygen measurements

Both isoalloxazine and alloxazine derivatives are interesting photosensitizers of singlet oxygen. However, there has been a lack of information on the singlet oxygen production by TMeAll. Therefore, in this work, investigations of singlet oxygen production by TMeAll were made by measurements of emission spectra centered at about 1270 nm and their kinetics. Figure 4S shows a representative ^1^O_2_ phosphorescence spectrum generated by **3a** in DCE. This emission is highly specific to the transition from singlet oxygen to triplet oxygen, which is described by: O_2_ (^1^Δ_g_) → O_2_ (^3^Σ_g_^−^).

It is known that singlet oxygen can be produced by oxygen quenching of photosensitizer singlet or triplet excited states. By comparing the fluorescence lifetimes of alloxazines (in the nanosecond range) to their triplet lifetimes (in the microsecond range), it was concluded that the alloxazines studied should only generate singlet oxygen in solution through their triplet state, since the probability of the alloxazine singlet state quenching should be very low due to its short fluorescence lifetime.

Table 2 lists the values of the quantum yields of photosensitized production of singlet oxygen (Ф_Δ_) by TMeAll in selected organic solvents, with perinaphthenone used as a reference. The data show that all derivatives are highly efficient photosensitizers of singlet oxygen. Comparing the data obtained in different organic solvents, a higher value of Ф_Δ_ was obtained for **3d** in MeOH (0.98) compared to ACN (0.86) and DCE (0.77). Lower values of Ф_Δ_ were observed for **3c** in MeOH (0.84) and for **3b** in DCE (0.65). These values of Ф_Δ_ are relatively high compared to other alloxazine derivatives, such as alloxazine **2f** (Ф_Δ_ = 0.36) and even monomethyl-alloxazine derivatives with a methyl group at C6 (Ф_Δ_ = 0.78), C7 (Ф_Δ_ = 0.69), C8 (Ф_Δ_ = 0.63) or C9 (Ф_Δ_ = 0.74), as well as the dimethyl derivative lumichrome (**2e**; (Ф_Δ_ = 0.73) in ACN^12^. Table 2 also lists the lifetimes of singlet oxygen generated by TMeAll in selected organic solvents (see also Fig. 4S). The decays recorded with λ_exc_ = 371 nm and λ_em_ = 1270 nm were described by a monoexponential function, and the measured lifetimes were typical of singlet oxygen in the solvents used about 80 µs in ACN, 70 µs 1,2-DCE, and 10 µs in MeOH and are consistent with reports in the literature^70^.

### Transient absorption spectra

In addition to the singlet state calculations, triplet state calculations were also performed, namely the S_0_ → T_i_ and T_1_ → T_i_ transition energies and oscillator strengths (Tables 3S and 4S). The lowest triplet excited state in all of the compounds examined has *π*,π* character.

**Figure 5:**
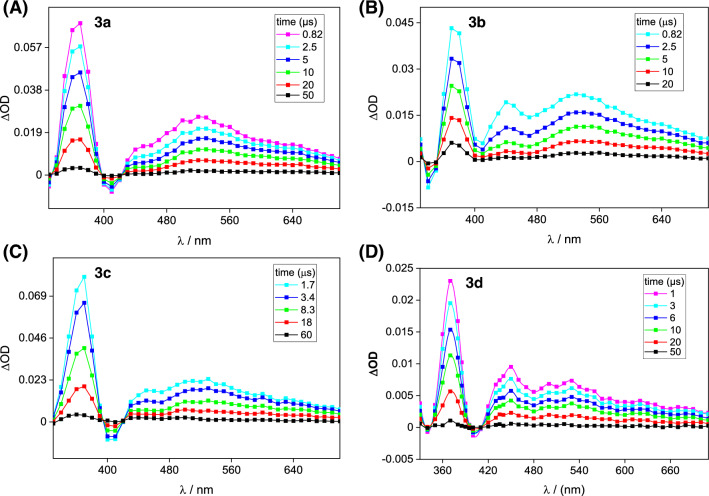
Experimental transient absorption spectra of tetramethylalloxazines (**A**-**3a**, **B**-**3b**, **C**-**3c**, **D**-**3d**) in acetonitrile excited at 355 nm, using OD_355_ = 0.5, 1.5 mJ/pulse, *l* = 1 cm.

The total spin operator S^2^ values for all the compounds studied were in the 2.02 range. The T_1_ → T_i_ excitation energies and transition intensities were determined for the optimized geometry of the lowest triplet state (T_1_). Figure 5S shows only those transitions that could be detected experimentally. The transitions were located at about 20.0 × 10^3^ cm^−1^ and about 24.5 × 10^3^ cm^−1^, and there was good agreement between calculated transitions for the dimethyl- and tetramethyl- analogues: for example, the two lowest energy transitions predicted for **3a** (and **2a**) are 20.4 × 10^3^ cm^−1^ (21.1 × 10^3^ cm^−1^) and 25.2 × 10^3^ cm^−1^ (25.6 × 10^3^ cm^−1^). Some lower-energy transitions of low oscillator strength were not observed experimentally due to limitations of the flash photolysis equipment used (see Table 4S).

The laser-flash-irradiation of solutions of TMeAll in ACN at 355 nm resulted in the formation of a transient species with a wide spectrum range of 350 to 700 nm, see Fig. 5. The experimental transient absorption spectra of all analyzed TMeAll are similar, with minor differences in the positions of the spectral maxima. The initial absorption spectrum of all studied compounds displayed a sharp maximum at 365 nm and broad absorption in the range of 420 to 700 nm, similar to the T-T absorption of the neutral triplet state reported for **2e**, monomethyl, dimethyl, and trimethyl-substituted alloxazines^23,26,71^. Negative absorbance was observed near 410 nm for **3b** and **3d**, and at about 340 nm for **3c**. The triplet lifetime of the studied compounds in acetonitrile was monitored, with the triplet lifetimes being in the range of microseconds (Fig. 5, Table 2). Analysis of the experimental triplet state decays indicates the presence of only one species. The transient spectrum ultimately decayed to baseline.

In terms of oxygen quenching of the triplet states, assuming that the concentration of oxygen in ACN at normal pressure is [O_2_]_lit_ = 2.42 mM,^72^, and 1.6 in DCE^73^, 2.15 in MeOH^72^, the rate constants determined for the process are on the order of magnitude $k_{{\text{T}}}^{{\left[ {{\text{O}}_{2} } \right]}}$ = 1.2 × 10^9^ M^−1^ s^−1^ or higher, the lifetimes of the triplet state lifetimes > 10 µs, and the triplet formed is almost entirely quenched by molecular oxygen at normal pressure ($P_{{\text{T}}}^{{\Delta }}$ = 0.96), $P_{{\text{T}}}^{{\Delta }} = \frac{{k_{{\text{T}}}^{{\left[ {{\text{O}}_{2} } \right]}} \times \left[ {{\text{O}}_{2} } \right]}}{{\left( {{\uptau }_{{\text{T}}}^{ - 1} + k_{{\text{T}}}^{{\left[ {{\text{O}}_{2} } \right]}} \times \left[ {{\text{O}}_{2} } \right]} \right)}}$ is a proportion of triplet being quenched by molecular oxygen at normal pressure.

The mechanism of energy transfer from the triplet state of **3a**, **3b, 3c** and **3d** to oxygen was also confirmed, as the value of $k_{{\text{T}}}^{{\left[ {{\text{O}}_{2} } \right]}}$/*k*_diff_ is quite close to one ninth^70^, where, $k_{{\text{T}}}^{{\left[ {{\text{O}}_{2} } \right]}}$ = rate constant of quenching the triplet state by oxygen and *k*_diff_ = rate constant for reaction limited by diffusion.

### Hemolytic activity

Hemocompatibility is a critical factor in the development of new compounds for biological research^74^. Red blood cells (RBC), which are the most abundant cells in human blood, are responsible for oxygen transport throughout the body^75^. Due to their crucial role in maintaining homeostasis, RBC are commonly used as a model system in in vitro studies^33^. In the current study, a hemolytic assay, which is a widely used test to assess the hematocompatibility/cytotoxicity of newly synthesized compounds, was performed to determine the potential toxicity of compounds **3a-3d** on RBC. Hemolytic activity of compounds **3a**-**3d** was evaluated at a concentration equal to 0.1 mg/mL in standard short-term incubation (1 h at 37 °C) and long-term incubation (24 h at 37 °C). The results obtained showed that after both short-term and long-term incubation, the degree of hemolysis for all compounds tested was in the range of 1.95% ± 0.78 to 3.49% ± 0.92 and was statistically similar to that obtained for control RBC, 2.03% ± 0.85 and 3.28% ± 0.67 (p > 0.05), respectively. It was considered that hemocompatible compounds used at a given concentration should not induce hemolysis greater than 5%. Therefore, based on the results obtained in this study, it can be concluded that all tested compounds used at a concentration of 0.1 mg/mL do not exhibit any adverse effects on the molecular structure of the lipid bilayer of the RBC membrane and do not increase its permeability to ions. These findings confirm that **3a**-**3d** are hemocompatible compounds and suggest their suitability for further analysis in fluorescence-lifetime imaging microscopy (FLIM).

### Fluorescence imaging in RBC under physiological and oxidative stress conditions

**Figure 6:**
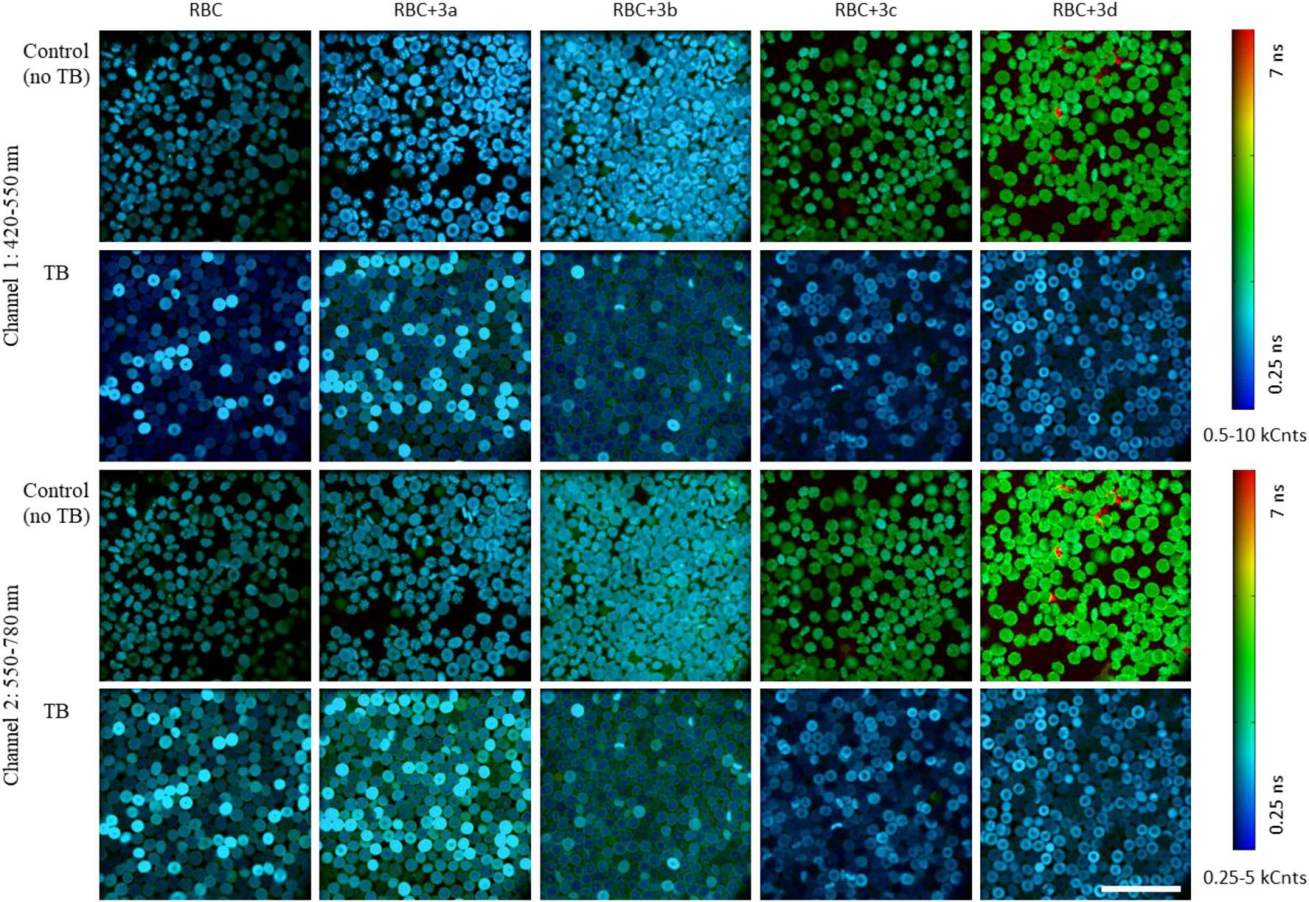
Representative FLIM images (exc 405 nm, 2 channels 420–550 nm and 550–780 nm) of **RBC, RBC + X** (where **X** mean preincubation with compounds **3a-3d** at 0.1 mg/1 mL concentration), and similar conditions but followed with TB incubation (oxidative stress, **RBC + TB** and **RBC + X + TB**). Color represents the average lifetime in the pixel (assuming monoexponential decay), while brightness corresponds to the total number of photons per pixel. All images were collected at identical instrument settings. The scalebar size is 50 µm.

In order to further investigate the effect and behavior of compounds **3a-3d** in RBC, fluorescence lifetime imaging microscopy (FLIM) analysis was performed. The lifetime data was collected in two different channels (channel 1: 420—550 nm and channel 2: 550—780 nm) at an excitation wavelength of 405 nm. Figure 6 shows the resulting representative images of the RBC, in which color encodes the average lifetime in the pixel while intensity shows the total number of photons per pixel.

An examination of the images indicates that fluorescence lifetimes are generally longer (greener) in case of cells exposed to **3c (RBC + 3c)** and even more so for **3d (RBC + 3d)**, compared to **RBC**, **RBC + 3a** and **RBC + 3b** in both channels (rows 1 and 3 in Fig. 6). In contrast, for RBC cells treated with TB (rows 2 and 4 in Fig. 6) the fluorescence lifetimes in both channels shorten for **RBC + 3c + TB** vs **RBC + 3c** and **RBC + 3d + TB** vs **RBC + 3d** (respectively columns 4 and 5 in Fig. 6) with less pronounced changes observed in case of the control **RBC + TB** (vs **RBC**), **RBC + 3a + TB** (vs **RBC + 3a**) and **RBC + 3b + TB** (vs **RBC + 3b)**. Interestingly, the images reveal three distinct cell phenotypes in terms of the intracellular distribution of relative fluorescence lifetimes, although most samples still exhibit mixed populations: a) uniform distribution across the cells (e.g. some brighter cells can be observed in **RBC + TB** – column 1 rows 2 and 4, and **RBC + 3a + TB** – column 2, rows 2 and 4, Fig. 6), b) donut-shaped with cells displaying a hollow center with thicker, more intense area around it, (observed in brighter cells of **RBC + 3c + TB** – column 4 rows 2 and 4, and **RBC + 3d + TB** – column 5 rows 2 and 4, Fig. 6) and c) shorter (more blue) lifetime values inside the cells with thin, usually longer lifetime (more green) rim area, possibly representing cell membrane (seen e.g. in both channels of **RBC + 3b + TB**—column 3 rows 2 and 4 in Fig. 6).

To further characterize effects of treatments (compounds and oxidative stress) on RBC, fluorescence lifetime parameters were extracted from the original images (at least three per condition). The data were processed by applying a bi-exponential fit and a subtraction of instrument response function. The resulting parameters, which represent the average data from at least three distinct images, are presented in Table 3.

**Table 3:** FLIM data for RBC cells or cells pretreated with compounds **3a-3d**, in physiological conditions and in oxidative stress induced by TB.

ch1: 420–550 nm	A_1_		A_2_		τ_1_ / ns		τ_2_ / ns		Sum of A		fraction τ_2_ / ns		w.mean** / ns	
	Mean	SEM	Mean	SEM	Mean	SEM	Mean	SEM	Mean	SEM	Mean	SEM	Mean	SEM
RBC	**2529**	390	**29**	4	**0.48**	0.00	**2.16**	0.28	**2986**	393	**0.01**	0.002	**0.50**	0.01
RBC + TB	**12,163**	819	**427**	15	**0.57**	0.00	**4.32**	0.31	**12,589**	811	**0.04**	0.004	**0.71**	0.03
RBC + 3a	**2572**	236	< 0*	n/a*	**0.50**	0.00	0.93*	0.84*	2572*	423	0.00*	n/a*	0.50*	0.00
RBC + TB + 3a	**14,304**	1508	**1046**	205	**0.60**	0.01	**4.75**	0.29	**15,350**	1319	**0.08**	0.025	**0.89**	0.06
RBC + 3b	**3710**	332	**161**	6	**0.52**	0.00	**3.31**	0.06	**3871**	327	**0.04**	0.005	**0.64**	0.01
RBC + TB + 3b	**10,910**	442	**448**	13	**0.56**	0.00	**5.36**	0.07	**11,358**	429	**0.04**	0.003	**0.75**	0.01
RBC + 3c	**1739**	244	**110**	9	**0.52**	0.00	**3.51**	0.03	**1849**	253	**0.06**	0.004	**0.71**	0.01
RBC + TB + 3c	**10,889**	430	**237**	17	**0.52**	0.00	**3.91**	0.05	**11,125**	429	**0.02**	0.002	**0.59**	0.01
RBC + 3d	**1601**	166	**411**	19	**0.72**	0.01	**4.23**	0.01	**2012**	185	**0.21**	0.010	**1.44**	0.04
RBC + TB + 3d	**13,558**	1255	**375**	29	**0.54**	0.00	**4.28**	0.06	**13,933**	1283	**0.03**	0.000	**0.64**	0.00

ch2: 550–780 nm	A_1_		A_2_		τ_1_ / ns		τ_2_ / ns		Sum of A		fraction τ_2_ / ns		w.mean**	
	Mean	SEM	Mean	SEM	Mean	SEM	Mean	SEM	Mean	SEM	Mean	SEM	Mean	SEM
RBC	**4639**	883	**266**	30	**0.42**	0.00	**1.64**	0.12	**5867**	908	**0.06**	0.007	**0.48**	0.01
RBC + TB	**18,763**	763	**778**	20	**0.54**	0.01	**3.64**	0.31	**19,541**	757	**0.04**	0.002	**0.67**	0.01
RBC + 3a	**4808**	666	**306**	12	**0.41**	0.00	**1.65**	0.08	**5114**	672	**0.06**	0.008	**0.48**	0.01
RBC + TB + 3a	**23,232**	2248	**1608**	341	**0.59**	0.01	**4.53**	0.21	**24,840**	1928	**0.08**	0.023	**0.86**	0.06
RBC + 3b	**7013**	799	**427**	7	**0.43**	0.00	**2.56**	0.02	**7440**	803	**0.06**	0.006	**0.56**	0.01
RBC + TB + 3b	**20,493**	945	**839**	23	**0.51**	0.00	**4.57**	0.03	**21,332**	924	**0.04**	0.003	**0.67**	0.01
RBC + 3c	**2679**	414	**180**	16	**0.44**	0.00	**2.75**	0.02	**2858**	430	**0.06**	0.005	**0.59**	0.02
RBC + TB + 3c	**17,196**	1021	**611**	25	**0.51**	0.00	**2.75**	0.04	**17,807**	1033	**0.03**	0.002	**0.58**	0.01
RBC + 3d	**2309**	262	**506**	18	**0.53**	0.01	**4.10**	0.02	**2816**	279	**0.18**	0.012	**1.18**	0.05
RBC + TB + 3d	**23,075**	1963	**758**	46	**0.53**	0.00	**3.37**	0.06	**23,833**	2009	**0.03**	0.001	**0.62**	0.00

Significance values are in bold.

*Bi-exponential fit of the data resulted in the amplitude values below 0 indicating a lack of a second component, therefore the calculated values take into consideration only τ_1_.

**w.mean is a weighted mean lifetime parameter τ calculated according to the equation (τ_1_*A_1_ + τ2*A_2_)/(A_1_ + A_2_).

A quick examination of the changes in the sum of amplitudes indicates a significant increase in fluorescence intensity following incubation with TB. This increase is observed across all conditions, including the control and all the compounds, in both channels. However, a more detailed analysis of the changes in lifetimes, τ_1_ and τ_2_, as well as their relative contributions (fraction of τ_2_) by separating the emission into two channels with different wavelength ranges, allows for a much better distinction between the different conditions.

The shorter lifetime parameter (τ_1_) values for cells not treated with TB show minimal differences in both channels. In channel 1, the τ_1_ values for **RBC** (0.48 ns) are similar to τ_1_ values for **RBC + 3a**, **RBC + 3b**, **RBC + 3c** and **RBC-3d** (0.50 ns—0.52 ns). In channel 2, the τ_1_ value = 0.42 ns for **RBC**, which lies within the range of values for other TB-untreated and compound-treated samples (0.41 ns—0.44 ns, respectively). Exposure of cells to TB cause an elongation of τ_1_ in both channels for **RBC + TB**, **RBC-3a + TB** and **RBC-3b + TB**. However, for **RBC-3c + TB**, an increase τ_1_ values compared to **RBC-3c** is observed only in channel 2, while in channel 1 τ_1_ remains constant at 0.52 ns (with a change from 0.44 ns to 0.51 ns from **RBC + 3c** to **RBC + 3c + TB**). **RBC + 3d**, on the other hand, differs from the above as it exhibits a longer τ_1_ in botch channels (0.72 ns vs 0.48 ns for **RBC** in channel 1 and 0.53 ns vs 0.42 ns for **RBC** in channel 2). Interestingly, addition of TB shortens its τ_1_ in channel 1 (from 0.72 ns for **RBC + 3d** to 0.54 ns in **RBC + 3d + TB**) while τ_1_ in channel 2 remains unaffected at 0.53 ns for both **RBC + 3d** and **RBC + 3d + TB**.

Examination of the value of τ_2_ and fraction of τ_2_ indicates broader diversity of these parameters between compounds than in the case of τ_1_. Specifically, fitting the FLIM data gives a bi-exponential fit in both channels for all conditions (control or compounds, with or without TB) with an exception of **RBC + 3a**, for which calculated A_2_ in channel 1 is negative suggesting lack of τ_2_. For the other compounds, the fraction of τ_2_ in channel 1 undergoes modest changes but in different directions. It increases from 1% for **RBC** to 4% for **RBC + TB**, and to 8% for **RBC + 3a + TB**, decreases from 6% for **RBC + 3c** to 2% for **RBC + 3c + TB**, and remains unchanged at 4% for **RBC + 3b** vs **RBC + 3b + TB**. In contrast, for **RBC + 3d**, fraction of τ_2_ in channel 1 is significantly higher at 21% and the value of τ_2_ is much longer at 4.23 ns. For **RBC + 3d + TB** (exposure to oxidative stress) the value of τ_2_ = 4.28 ns (largely similar to **RBC + 3d**) but its contribution drops to 3% amounting to a significant shortening of average fluorescence lifetime (weighted lifetime decreases from 1.44 ns for **RBC + 3d** to 0.64 ns for **RBC + 3d + TB**). Similar differences are observed when examining τ_2_ in channel 2 for **RBC + 3d** and **RBC + 3d + TB** compared to the other conditions. The contribution of τ_2_ drops from 18 to 3% and the τ_2_ value change from 4.10 ns to 3.37 ns for **RBC + 3d** vs **RBC + 3d + TB**. For all other conditions (control and compounds), the fraction of τ_2_ in channel 2 remains at 6% at physiological conditions, with values at 1.64 ns < 1.65 ns < 2.56 ns < 2.75 ns < 4.10 ns respectively for **RBC + 3a**, **RBC + 3b**, **RBC + 3c**, **RBC + 3d** and **RBC**. TB treatment decreases the fraction of τ_2_ to 4% for **RBC + TB** and **RBC + 3b + TB**, and to 3% for **RBC + 3c + TB**, while for **RBC + 3a + TB** it increases to 8%. The τ_2_ values lengthen for **RBC + TB** (2.64 ns), **RBC + 3a + TB** (4.53 ns) and **RBC + 3b + TB** (4.57 ns), while remaining unaffected for **RBC + 3c + TB** in comparison to **RBC + 3c**. In summary, extracted fluorescence lifetime parameters for both channels support in a more detailed way the trends exhibited by the images in Fig. 6. They indicate that compounds **3c** and **3d** induce the most noticeable changes in these parameters in RBC cells.

**Figure 7:**
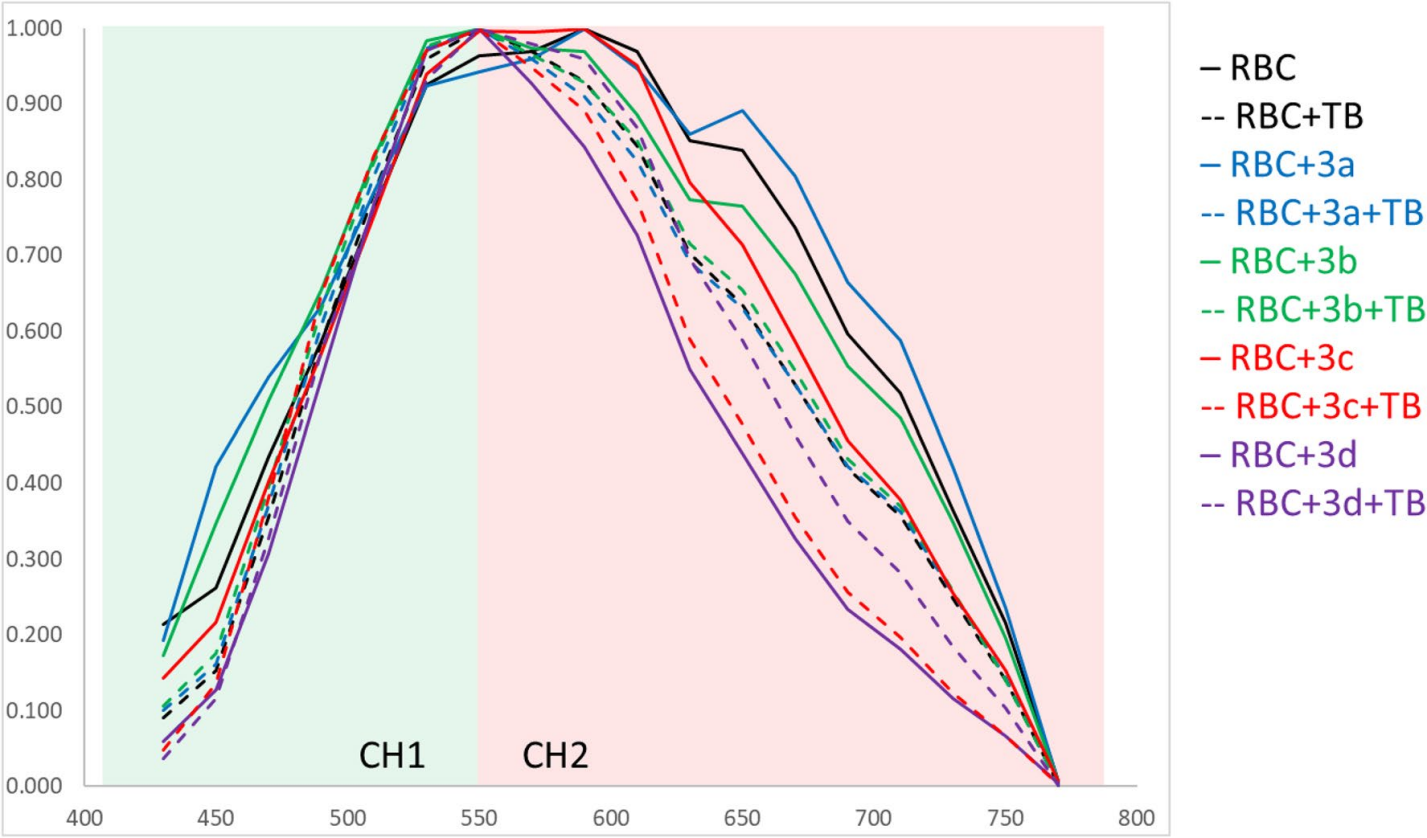
Normalized fluorescence emission spectra in cells collected sequentially with 20 nm rolling detection window in 20 nm steps from 420 to 780 nm for fixed 405 nm excitation. Values averaged over two spectra per compound-treated samples (**RBC + X** and **RBC + X + TB** with **X** being compounds **3a-3d**) and four spectra for **RBC** and **RBC + TB.**

To complement the fluorescence lifetime data, fluorescence spectra of the cells were collected by taking images sequentially with different emission ranges (20 nm steps) using the same excitation wavelength (405 nm) for cells at all of the above-mentioned conditions. The obtained spectra were subsequently normalized (Fig. 7), enabling a comparison of the spectral shape. In line with our previous report^36^, the normalized spectra reveal the presence of two primary emission regions centered around 550 nm (likely associated with flavin-like compounds) and 650 nm (presumably of endogenous intracellular origin).

The spectra of **RBC + 3a** (solid blue line, Fig. 7) and **RBC + 3b**, (solid green line) exhibit similar shapes to the one of **RBC** (solid black line) with the 650 nm band most prominent in order of **RBC + 3a > RBC > RBC + 3b.** The spectrum induced by **3c** (**RBC + 3c**, solid red line) shows a less pronounced 650 nm band and becomes similar to the spectrum of **RBC + TB** (black dashed line). Interestingly, for **RBC + 3d** (solid violet line), the 650 nm band almost disappears, suggesting a significantly different influence of **3d** on RBC in comparison to all other compounds. Exposure of cells to oxidative stress (dashed line spectra in Fig. 7) leads to spectra of **RBC + 3a + TB** and **RBC + 3b + TB** (dashed blue and dashed green lines, respectively) being almost indistinguishable in shape from **RBC + TB** (dashed black line). Oxidative stress also seems to diminish the previously distinct impact of **3d** on the spectral shape (**RBC + 3d + TB**, dashed violet line) as it also resembles **RBC + TB**. Spectra comparison shows a reverse direction of the TB-induced spectral change in **RBC + 3d + TB** vs **RBC + 3d** in comparison to samples with compounds. On the other hand, TB-treatment results in a decrease in the relative contribution of the 650 nm band in **RBC + 3c + TB** (dashed red line) giving the most distinct spectrum among all TB-treated samples (dashed lines) and resembling **RBC + 3d**, (solid violet line). These spectral changes support the conclusion of a distinct impact of compounds **3c** and **3d** on RBC.

## Conclusions

The spectral and photophysical properties of certain tetramethylalloxazines (TMeAll) were studied and compared with data from other alloxazine derivatives. This class of compounds typically have the *π*,π* state as the lowest lying singlet state, but the n,π* states are accompanied with the *π*,π* character and in some cases, such as the **3b** derivative, an inversion occurs where the n,π* states become the lowest energy singlet state. Comparison with data from other alloxazine derivatives reveals that the presence of a methyl group at the C8 position often leads to this inversion. In contrast, methyl substitution at the N1 and N3 positions has little impact on the spectral and photophysical properties of alloxazines.

In addition, data from transient absorption and singlet oxygen measurements have been collected to demonstrate that the TMeAll are highly effective photosensitizers for singlet oxygen, regardless of the solvent used. It is worth noting that in some cases, the sum of Φ_F_ + Φ_Δ_ is close to unity, and in all cases is very high.

**2e** (7,8-dimethylalloxazine), a closely related analog of the compounds studied, along with **1a** and 3-methyl- tetraacetylriboflavin, has already demonstrated non-mutagenic, non-genotoxic, and non-clastogenic properties^76^. These compounds are used in photodynamic therapy^77–79^ and in antibacterial photodynamic therapy^5^. From a photophysics standpoint, it is reasonable to assume that the compounds being studied will have similar applications due to their higher quantum efficiency in singlet oxygen formation, as well as other spectral and photophysical properties.

These findings are important due to their potential applications in catalysis and photooxygenations. For example, a procedure for sulfoxidation with riboflavin tetraacetate has been developed, demonstrating a practical application of flavins in oxygenation reactions using singlet oxygen^80,81^. We have shown in the present work that TMeAll can generate singlet oxygen with high yield and therefore represent a convenient collection of ^1^O_2_ photosensitizers for studies that require singlet oxygen. As these TMeAll can generate singlet oxygen with high yield, they represent a collection of convenient ^1^O_2_ photosensitizers. Moreover, a more thorough knowledge about the triplet state properties of these sensitizers (viz. *E*_T_, *τ*_T_, Φ_T_) can help guide future design and define the maximum performance of the sensitizers.

All investigated compounds showed no hemolytic activity against human red blood cells at the concentration tested. Therefore, they can be considered safe for use in any biomedical application at this concentration and may have potential as redox-sensitive agents.

The analysis of fluorescence lifetime imaging microscopy (FLIM) in two different channels and combined with fluorescence spectra measurements revealed differences in the impact of **3a-3d** on RBC. Furthermore, it highlighted variations in RBC response to oxidative stress, with compounds **3c** and **3d** exerting the most distinct effects. We have developed and validated a novel, multichannel FLIM protocol to generate unique and diverse datasets. This protocol enables us to distinguish the redox/fluorescent effects of flavin analogs on cellular models. As evident, there are limited studies on the application of FLIM in RBC studies, particularly in differentiating the redox/fluorescent effects of flavin analogs or other probes on RBC. There are several reasons for this, including the weak emission of RBCs under normal conditions. As a result, research on RBC, both in normal conditions and oxidative stress conditions, has primarily relied on optical microscopy. In our work, we believe that we have overcome this barrier and introduced FLIM as a method for studying RBC under oxidative stress conditions. The FLIM protocol and resulting data can serve as a tool to guide the future design and development of flavin-based molecular tools. These tools may include sensors for detecting redox changes in cells, antioxidants, and singlet-oxygen generators for PDT. Our objective was not to validate new PDT tools, but rather to demonstrate the diverse parameters that can be obtained using new FLIM methodology.

### Supplementary Information

Supplementary Information.

## Data Availability

The datasets generated during the current study are available from the corresponding author on reasonable request.
