# Supplementary material for: Tetramethylalloxazines as efficient singlet oxygen photosensitizers and potential redox-sensitive agents
Source: Sci Rep. 2023 Aug 17;13:13426. doi: 10.1038/s41598-023-40536-4 (PMC10435492; doi:10.1038/s41598-023-40536-4)
Supplement: Supplementary file 1 — Supplementary Information. [file 41598_2023_40536_MOESM1_ESM.pdf]

Supplementary data for

Tetramethylalloxazines as efficient singlet oxygen photosensitizers and potential redox-sensitive agent

Anna Golczak[a], Dorota Prukała[a], Ewa Sikorska[b], Mateusz Gierszewski[c], Volodymyr Cherkas[d], Dorota Kwiatek[d], Adam Kubiak[a], Naisargi Varma[a], Tomasz Pędziński[a], Shaun Murphree[e], Radek Cibulka[f,*], Lucyna Mrówczyńska[g,*], Jacek Lukasz Kolanowski[d,*] and Marek Sikorski[a,*]

[a]Faculty of Chemistry, Adam Mickiewicz University, Uniwersytetu Poznańskiego 8, 61-614 Poznań, Poland, E–Mail: sikorski@amu.edu.pl (M.S.)
[b]Poznań University of Economics and Business, Al. Niepodległości 10, 61-875 Poznań, Poland
[c]Faculty of Physics, Adam Mickiewicz University, Uniwersytetu Poznańskiego 2, 61-614 Poznań, Poland
[d]Institute of Bioorganic Chemistry, Polish Academy of Sciences, Noskowskiego 12/14, 61-704, Poznań, Poland, E-Mail: jacek.kolanowski@ibch.poznan.pl (J.L.K.)
[e]Department of Chemistry, Allegheny College, 520 N. Main Street, Meadville, PA, USA
[f]Department of Organic Chemistry, University of Chemistry and Technology, Prague, Technicka 5, 16628 Prague 6, Czech Republic, E-Mail: radek.cibulka@vscht.cz (RC).
[g]Faculty of Biology, Adam Mickiewicz University, Uniwersytetu Poznańskiego 6, 61-614 Poznań, Poland, E–Mail: lumro@amu.edu.pl (L.M.)

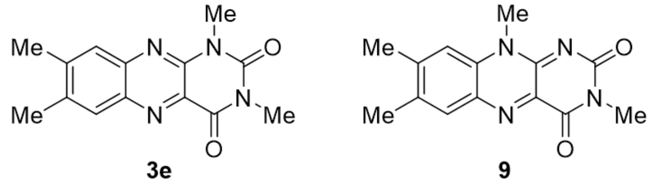

condition	$\bar{\nu}_{min} \cdot 10^{-3}$ cm	
	**3e**	**9**
1,4-dioxane	26.18	22.5
gas-phase	26.5	22.9
calculated	27.5	24.5

Figure 1S. Experimental maxima and calculated energy of $S_0 \rightarrow S_i$ of lowest π,π^* transitions for 1,3-dimethyllumichrome (**3e**) and 3,7,8,10-tetramethylisoalloxazine (**9**). Data taken from reference [1].

3b **3c**

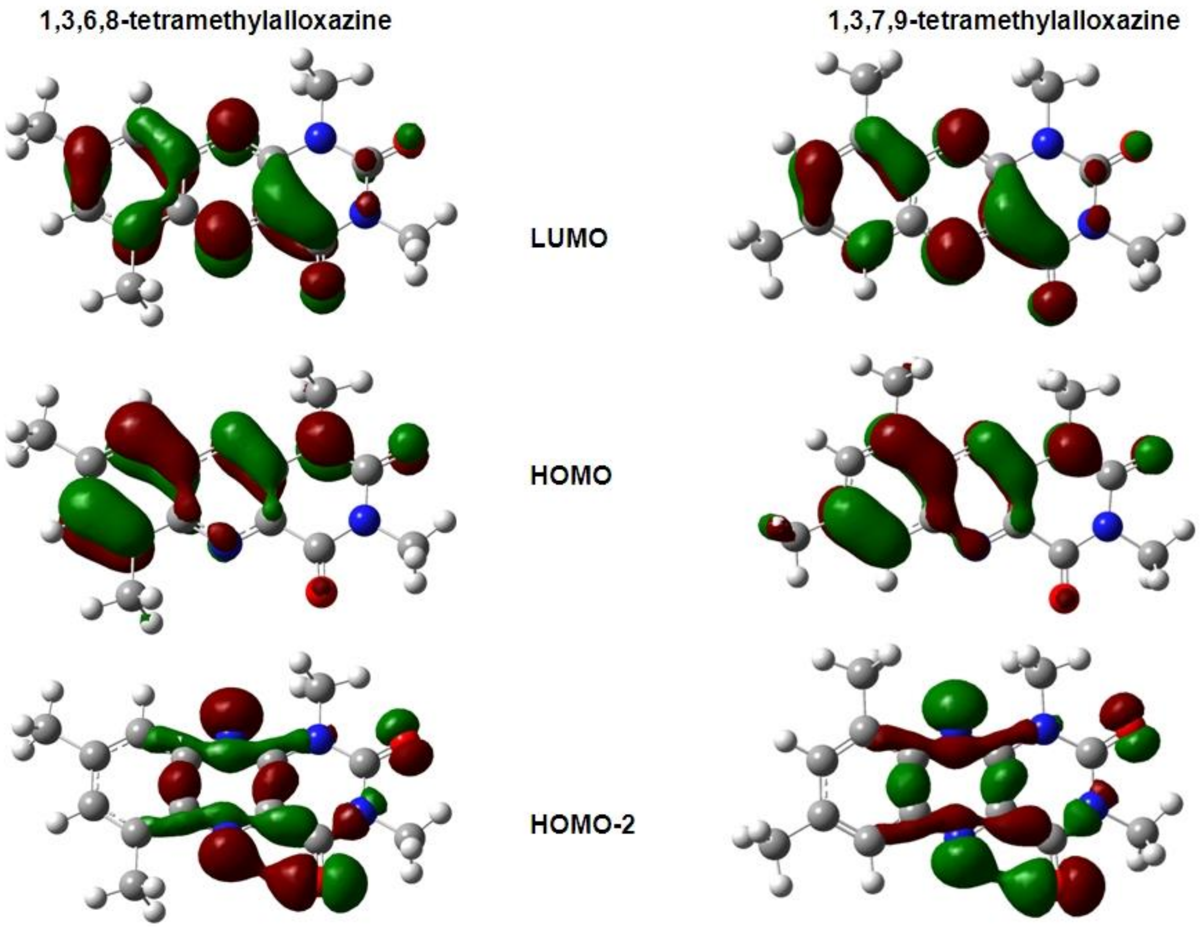

Figure 2S. The shape of the highest occupied molecular orbitals (HOMO) and the lowest unoccupied molecular orbitals (LUMO) of **3b** and **3c**, mainly involved in the lowest singlet-singlet transitions. The isosurfaces correspond to the wave function value of ±0.02.

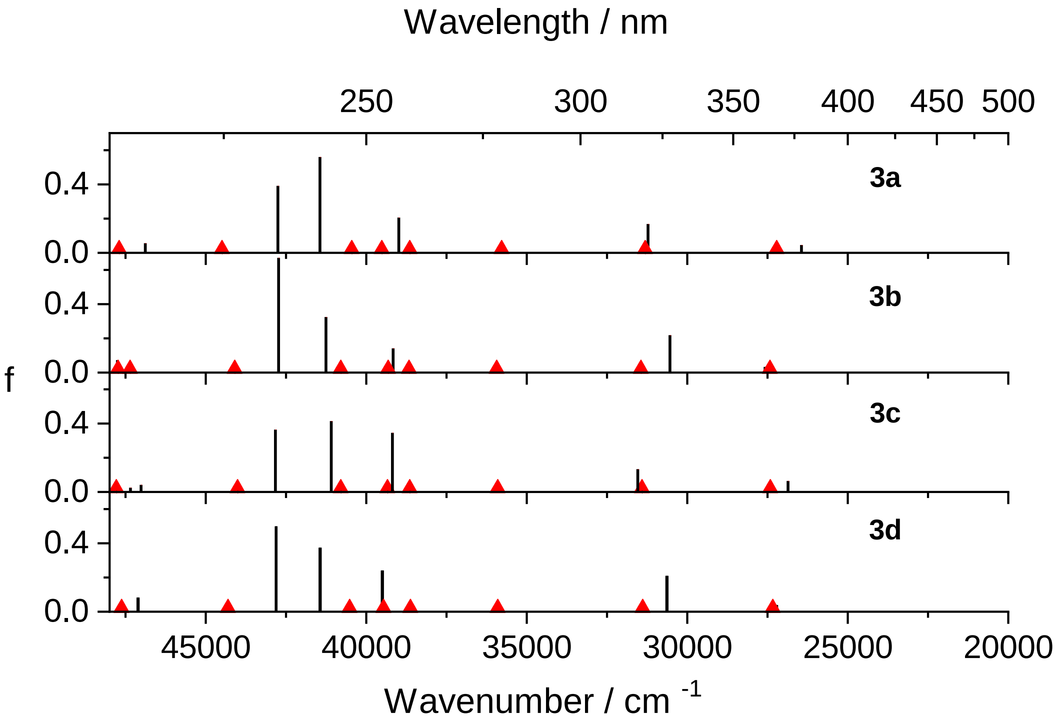

Figure 3S. Predicted $S_0 \rightarrow S_i$ transitions in TMeAll obtained in the DFT (B3LYP/6-31G(d)) calculations. Transition energies and oscillator strengths (*f*) are indicated by solid vertical bars. Triangles mark the weak n,π* transitions.

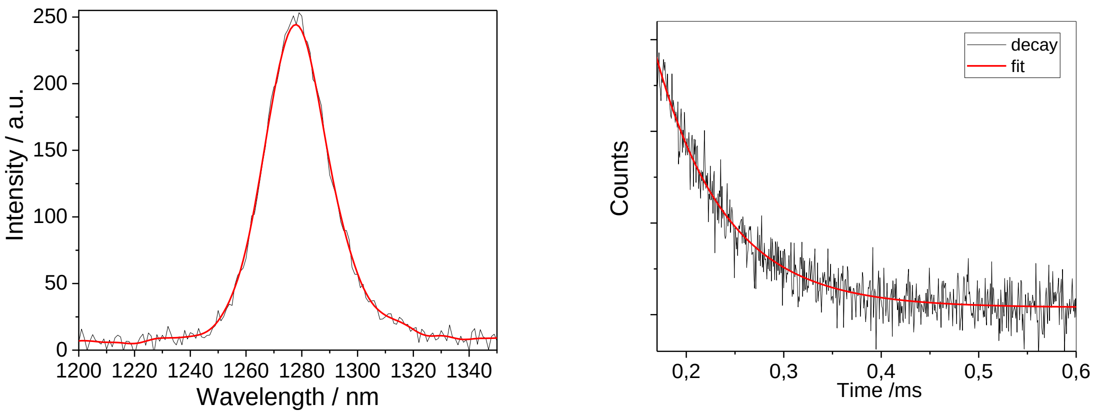

Figure 4S. Phosphorescence spectrum of singlet oxygen (peaking at λ_{max} ~ 1270 nm) generated by **3a** in DCE (λ_{exc} = 378 nm) (on the left). Decay of singlet oxygen phosphorescence (λ_{exc} = 378 nm, λ_{em} = 1270 nm) produced by **3a**, τ_{Δ} = 67μs (on the right).

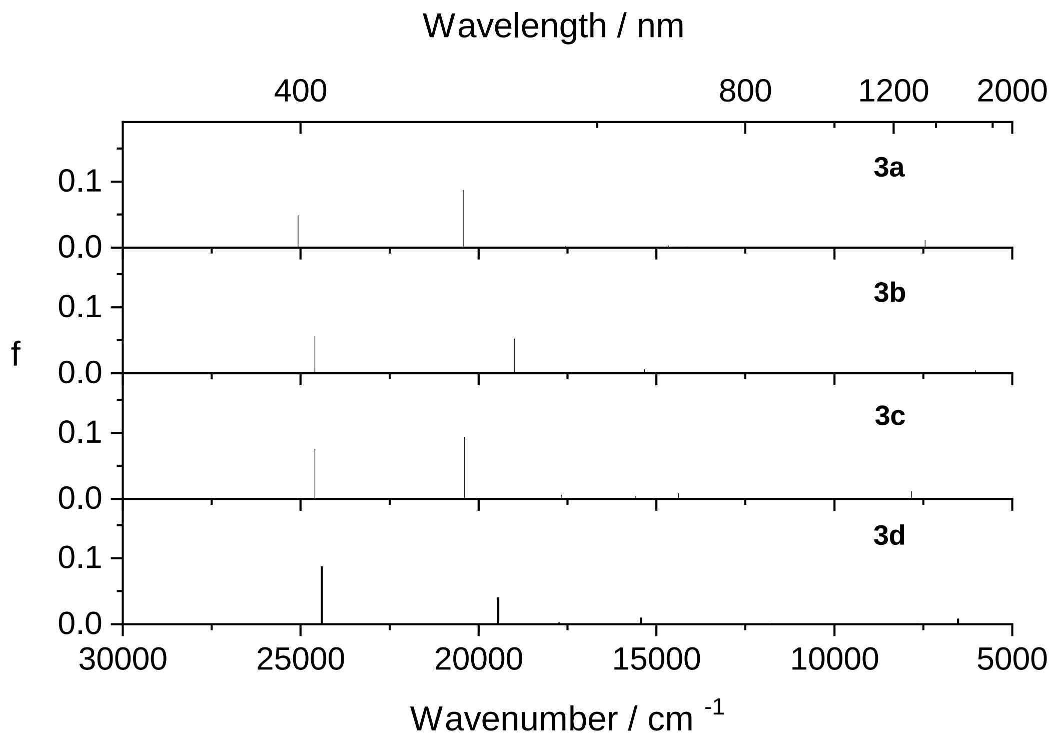

Figure 5S. Predicted $T_1 \rightarrow T_i$ transitions in TMeAll obtained in the DFT (UB3LYP/6-31G(d)) calculations; calculated triplet excitation energies, *E*, starting from the lowest triplet state and the corresponding oscillator strengths, *f*.

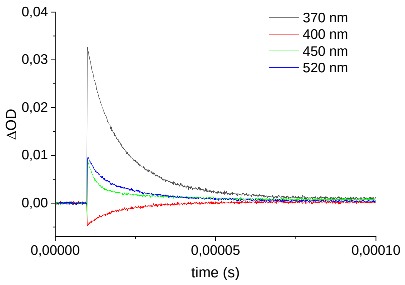

Figure 6S. Kinetics determined by laser-flash-irradiation of solutions of 3a in ACN at 355 nm excitation. The signals were recorded at the 370, 400, 450 and 520 nm.

3a

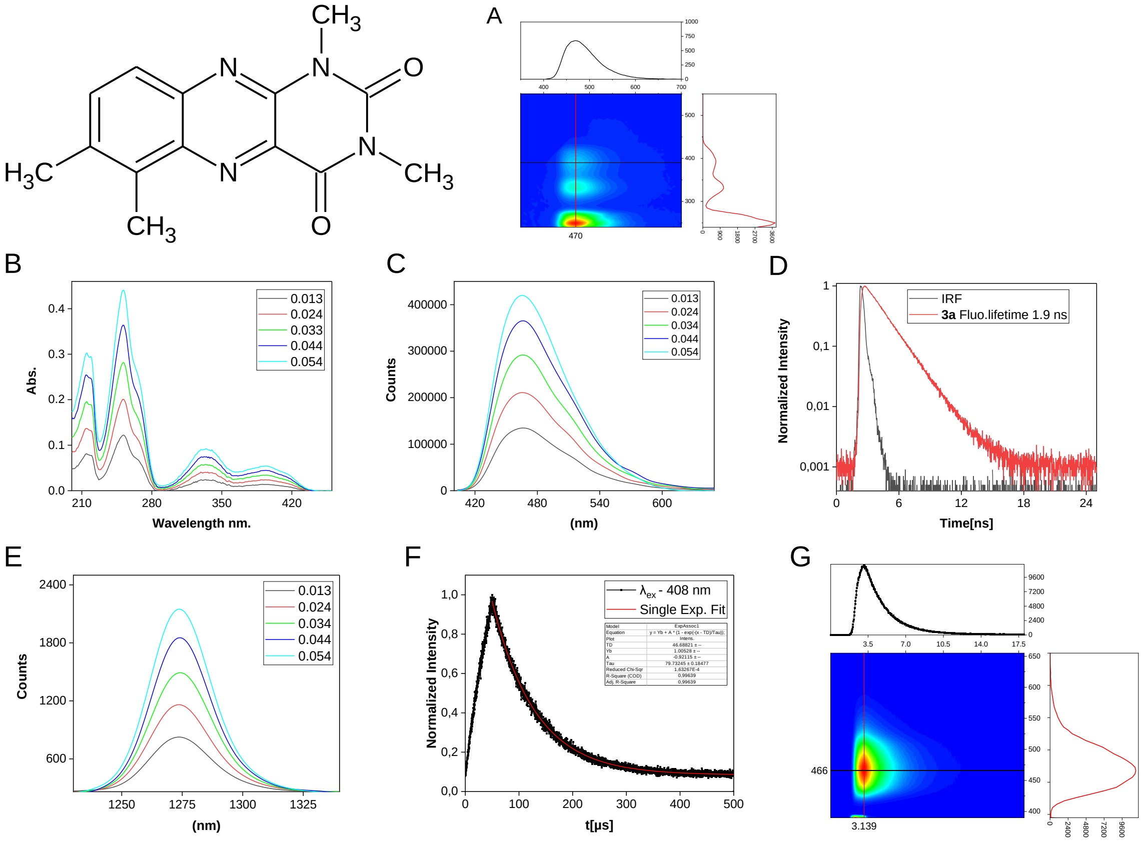

Figure 7S. Spectral and photophysical properties of **3a** in acetonitrile.

3b

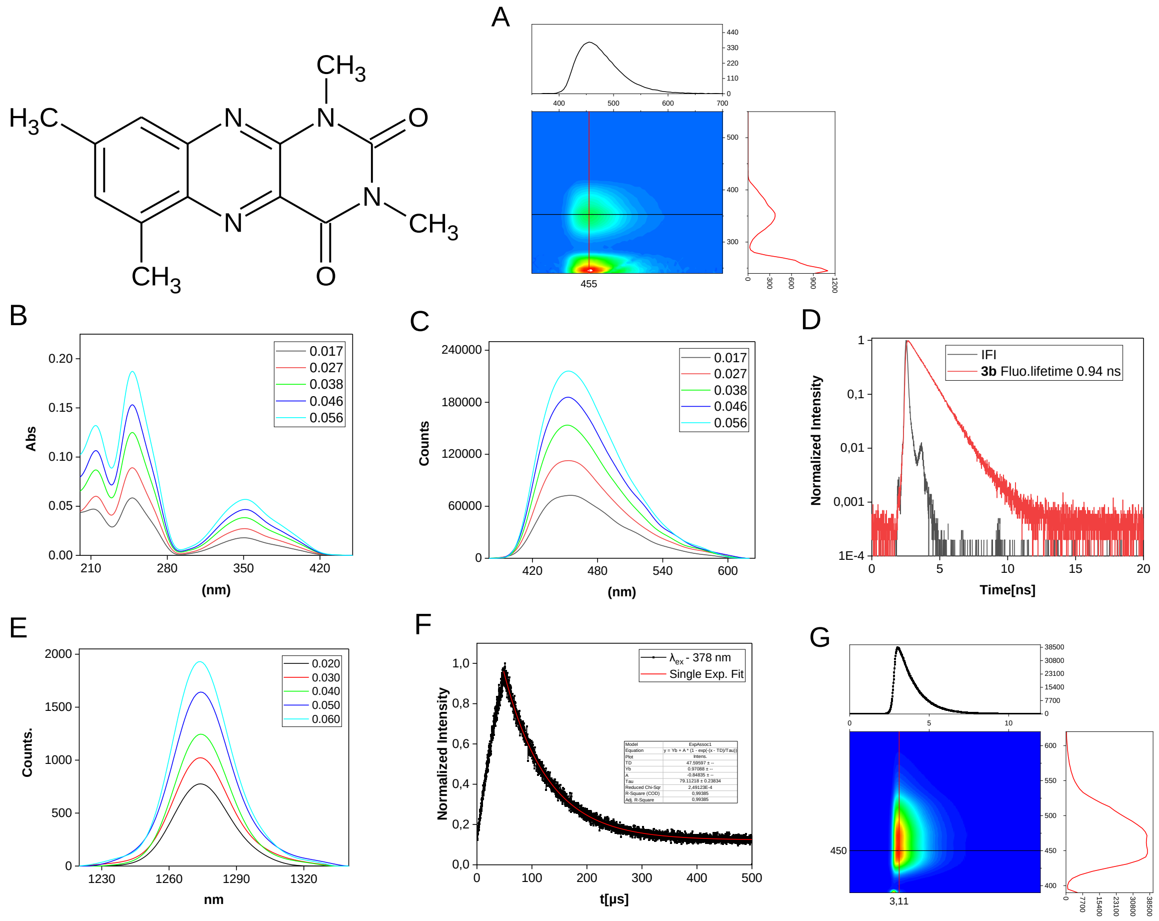

Figure 8S. Spectral and photophysical properties of **3b** in acetonitrile.

3c

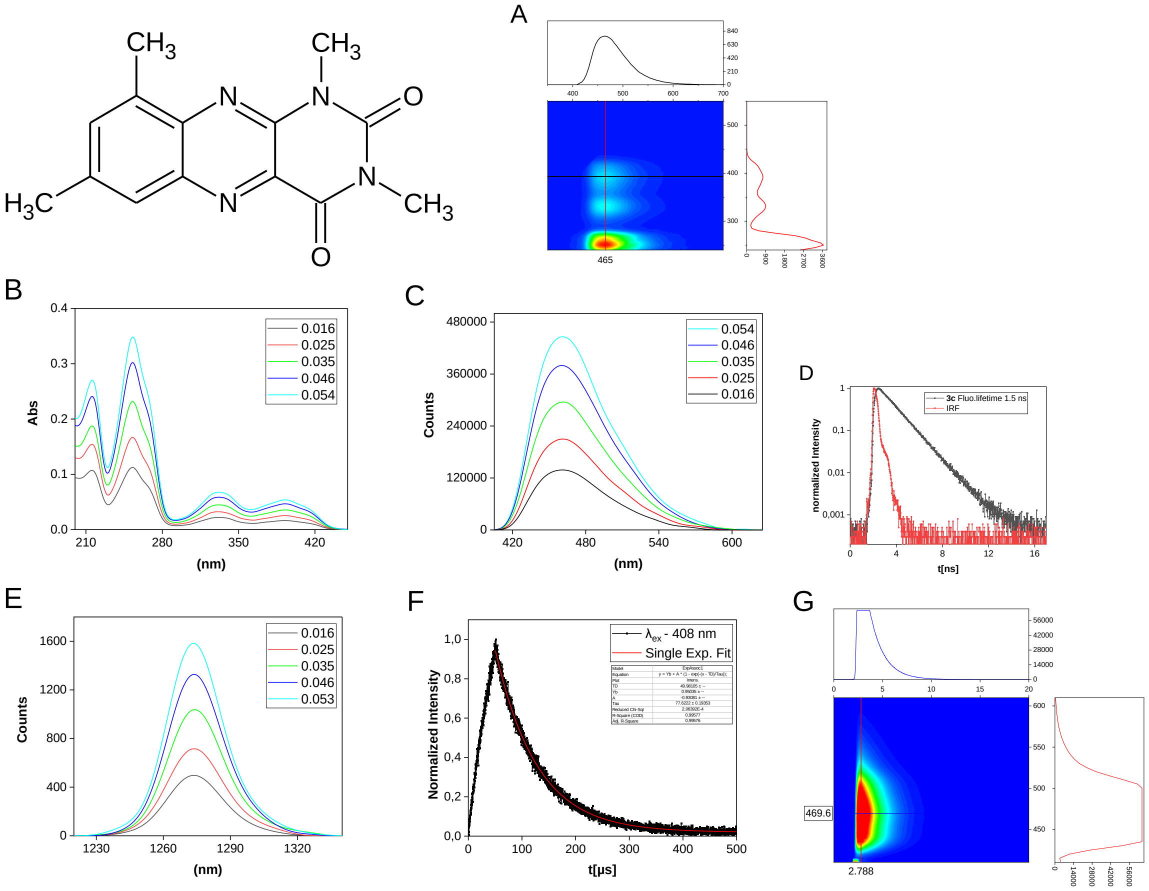

Figure 9S. Spectral and photophysical properties of **3c** in acetonitrile.

3d

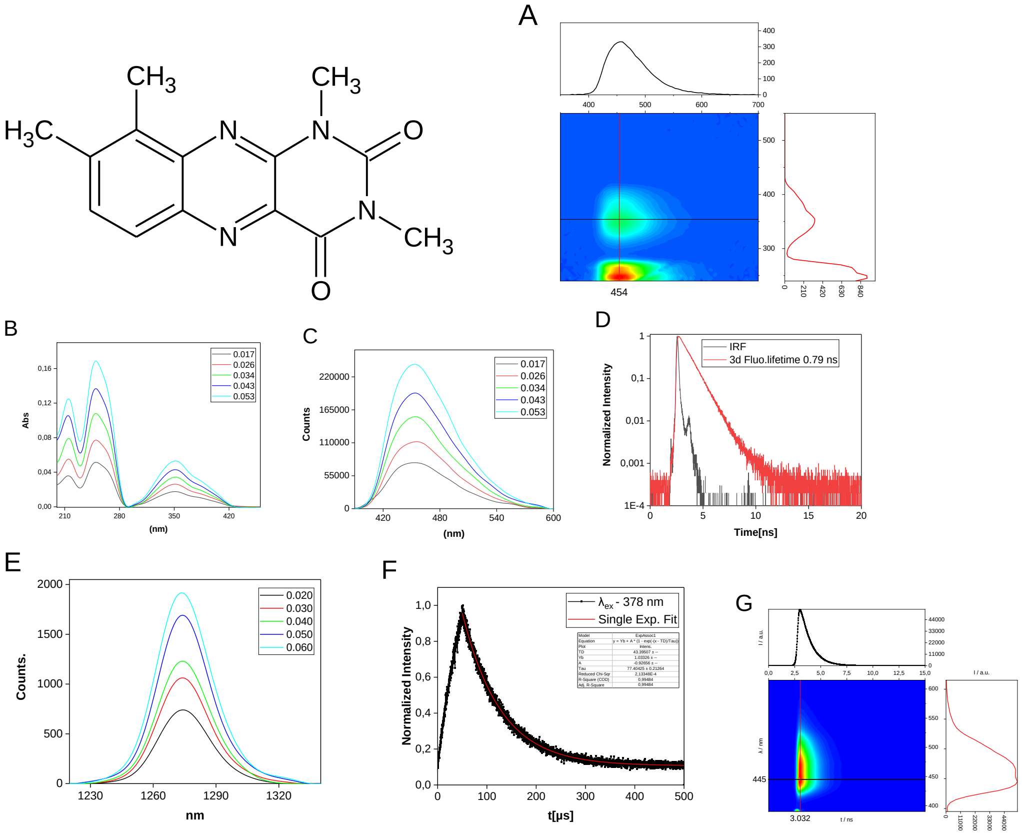

Figure 10S. Spectral and photophysical properties of **3d** in acetonitrile.

Experimental Method: -

Spectral and photophysical properties of studied compound in acetonitrile; Panel **(A)** fully corrected emission excitation matrix (EEM) recorded on Aqualog-UV-800 HORIBA Scientific/Jobin Yvon Technology with the A-TEEMs^{TM} (absorbance-transmission and fluorescence excitation-emission matrix) method. The panels on top and on the right side of the EEM represent emission and florescence excitation spectra for selected wavelengths of excitation and emission, respectively. Panel **(B)** Absorption spectra recorded on a UV-2550-230V/SHIMADZU spectrophotometer. **(C)** Fluorescence Spectra recorded on a Jobin Yvon-Spex Fluorolog 3-221 spectrofluorometer with a UV-PMT module, slit width 1/1nm, integration time 0.1 s, A Xe lamp with a monochromator was used for excitation. **(D)** Typical fluorescence decays of compound studied in acetonitrile, measured on fluorescence lifetime spectrometer/ Fluo Time 300-Pico Quant, using LDH-405 diode laser operated at 408 nm for compound **3a, 3c** and LDH-375 diode laser operated at 378 nm for compound **3b** and **3d**. **(E)** Singlet oxygen-phosphorescence spectra was recorded on fluorescence lifetime spectrometer/ FluoTime 300-Pico Quant, sensitive between 1200nm to 1350nm NIR rang (λ_{em}=1270 nm) having HAMAMATSU- NIR-PMT detector-module controller Max 800V, using a lamp as an excitation source (λ_{Ex}-375 nm for **3b**, **3d** and λ_{Ex}-408 nm for **3a**, **3c**), excitation slit width 5 nm, integration time 0.3 s. **(F)** Typical singlet oxygen decays of studied compound in acetonitrile, were measured using a LDH-405 diode laser operated at 408nm for compound **3a, 3c** and LDH-375 diode laser operated at 378 nm for compound **3b, 3d** at λ =1270 nm using FluoTime 300-

PicoQuant instrument. **(G)** Time resolved emission spectra-TRES 3D-Decay were recorded on fluorescence lifetime spectrometer / Fluo Time 300-Pico Quant using a LDH-405 diode laser operated at 408 nm for compound **3a**, **3c** and LDH-375 diode laser operated at 378 nm for compound **3b**, **3d.** (The numerical data received using single exponential fit are presented in Table)

Table 1S. The lowest predicted (B3LYP/6-31G(d)) $S_0 \rightarrow S_i$ excitation energies with their corresponding oscillator strengths, *f* for dimethyl-substituted derivatives of alloxazine. Data from Ref. [2].

	**2a**		**2b**		**2c**		**2d**	
$S_0 \rightarrow S_i$	$E \times 10^{-3}$ / cm^{-1}	*f*	$E \times 10^{-3}$ / cm^{-1}	*f*	$E \times 10^{-3}$ / cm^{-1}	*f*	$E \times 10^{-3}$ / cm^{-1}	*F*
$\rightarrow S_1$	26.4	0.038	27.6[b]	0.002	26.9	0.054	27.2	0.027
$\rightarrow S_2$	27.4[b]	0.002	27.6	0.025	27.5[b]	0.002	27.5[b]	0.002
$\rightarrow S_3$	31.6	0.164	30.9	0.217	31.8	0.135	30.9	0.206
$\rightarrow S_4$	31.9[b]	<0.001	32.0[b]	<0.001	32.0[b]	<0.001	31.9[b]	<0.001
$\rightarrow S_5$	38.6	0.024	38.7[b]	0.008	38.6	0.044	38.6	0.011

b - n,π* state, otherwise π,π* state

Table 2S. Spectroscopic and photophysical data for the singlet states of dimethyl-substituted derivatives of alloxazine in methanolic solution. Data from Ref. [2].

Compound	λ_2 / nm	λ_1 / nm	λ_F / nm	Φ_F	τ_F / ns	k_r / 10^8 s^{-1})	Σk_{nr} / 10^8 s^{-1})
**2a**	335	391	486	0.075	4.2	0.18	2.2
**2b**	352	-	475	0.034	2.28	0.15	4.2
**2c**	333	389	476	0.10	3.7	0.27	2.4
**2d**	354	383	473	0.039	2.41	0.16	4.0

Table 3S. The lowest predicted (B3LYP /6-31G(d)) $S_0 \rightarrow T_i$ excitation energies of TMeAll with their corresponding oscillator strengths, *f*.

	**3a**		**3b**		**3c**		**3d**	
$S_0 \rightarrow T_i$	$E \times 10^{-3}$ / cm^{-1}	*f*	$E \times 10^{-3}$ / cm^{-1}	*f*	$E \times 10^{-3}$ / cm^{-1}	*f*	$E \times 10^{-3}$ / cm^{-1}	*F*
$\rightarrow T_1$	19.3	0	20.4	0	19.8	0	19.7	0
$\rightarrow T_2$	23.0	0	22.7	0	23.1	0	22.8	0
$\rightarrow T_3$	23.3	0	23.5	0	23.4	0	23.4	0
$\rightarrow T_4$	28.3	0	28.4	0	28.4	0	28.3	0
$\rightarrow T_5$	30.4	0	30.7	0	30.0	0	30.6	0
$\rightarrow T_6$	31.2	0	31.6	0	31.3	0	31.5	0
$\rightarrow T_7$	33.4	0	33.6	0	34.2	0	34.1	0
$\rightarrow T_8$	34.9	0	35.2	0	35.1	0	34.9	0
$\rightarrow T_9$	36.6	0	35.7	0	36.6	0	35.7	0
$\rightarrow T_{10}$	36.8	0	37.1	0	36.8	0	36.8	0

Table 4S. The lowest predicted (UB3LYP/6-31G(d)) $T_1 \rightarrow T_i$ excitation energies of TMeAll with their corresponding oscillator strengths, *f*.

	**3a**[a]		**3b**[b]		**3c**[c]		**3d**[d]	
$T_1 \rightarrow T_i$	$E\times 10^{-3}$ / cm^{-1}	*f*	$E\times 10^{-3}$ / cm^{-1}	*f*	$E\times 10^{-3}$ / cm^{-1}	*f*	$E\times 10^{-3}$ / cm^{-1}	*f*
$\rightarrow T_2$	7.4	0.011	6.0	0.005	7.8	0.012	6.5	0.009
$\rightarrow T_3$	7.8	0	7.6	0	7.9	0	7.7	0
$\rightarrow T_4$	12.1	0	11.4	0	12.4	0	11.8	0
$\rightarrow T_5$	14.3	0.003	14.3	0.001	14.4	0.008	14.7	<0.001
$\rightarrow T_6$	15.3	0.003	15.3	0.006	15.6	0.004	15.47	0.01
$\rightarrow T_7$	17.4	0.012	18.0	<0.001	17.7	0.006	17.77	0.003
$\rightarrow T_8$	18.9	<0.001	18.9	<0.001	19.1	<0.001	18.7	<0.001
$\rightarrow T_9$	20.3	0	19.0	0.052	20.4	0.094	19.47	0.041
$\rightarrow T_{10}$	20.4	0.112	19.5	<0.001	20.6	0	20.0	0
$\rightarrow T_{11}$	25.2	0.042	24.6	0.056	24.6	0.075	24.4	0.088

a - T_1 = - 911,361491 Hartree/part., S**2=2,022384
b - T_1 = - 911,361186407 Hartree/part., S**2=2,0216
c - T_1 = - 911,3630791 Hartree/part., S**2=2,02013
d - T_1 = - 911,3591616 Hartree/part., S**2=2,020815

[1] M. Bruszyńska, E. Sikorska, A. Komasa, I. Khmelinskii, L.F.V. Ferreira, J. Hernando, J. Karolczak, M. Kubicki, J.L. Bourdelande, M. Sikorski, Electronic structure and spectral properties of selected trimethyl-alloxazines: Combined experimental and DFT study, Chem. Phys. 361 (2009) 83-93.
[2] M. Sikorski, D. Prukała, M. Insińska-Rak, I. Khmelinskii, D.R. Worrall, S.L. Williams, J. Hernando, J.L. Bourdelande, J. Koput, E. Sikorska, Spectroscopy and photophysics of dimethyl-substituted alloxazines, J. Photochem. Photobiol., A 200 (2008) 148-160.
